# Phenyl Radical-Mediated Fluorogenic Cyclization for Specific Detection of Peroxynitrite

**DOI:** 10.1021/acs.analchem.4c06983

**Published:** 2025-03-27

**Authors:** Aleksandra Grzelakowska, Radosław Podsiadły, Jacek Zielonka

**Affiliations:** † Institute of Polymer and Dye Technology, Faculty of Chemistry, Lodz University of Technology, Stefanowskiego 16, Lodz 90-537, Poland; ‡ Department of Biophysics, Medical College of Wisconsin, 8701 Watertown Plank Road, Milwaukee, Wisconsin 53226, United States

## Abstract

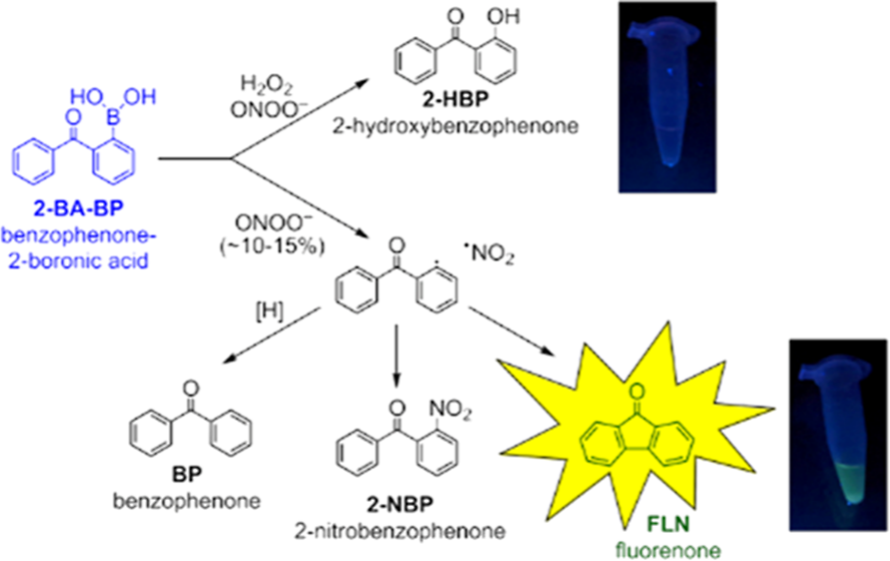

Peroxynitrite (ONOO^–^), a biological oxidizing and nitrating species responsible for post-translational modification of cellular proteins, has been implicated in numerous pathologies carrying an inflammatory component. Specific detection of ONOO^–^ in biological systems remains a challenge, and boronates are regarded as the most promising class of probes for the detection and quantitation of ONOO^–^. Oxidation of boronate probes by ONOO^–^ results in the formation of minor ONOO^–^-specific products via a pathway involving a phenyl radical-type intermediate, in addition to the major phenolic product. Here, we report fluorogenic cyclization of the phenyl-type radical formed during oxidation of a boronate probe by ONOO^–^, with the production of a fluorescent product, and we propose a new approach for the specific detection of ONOO^–^ based on this observation. We characterized the kinetics and stoichiometry of the reaction of benzophenone-2-boronic acid with ONOO^–^ and identified 2-hydroxybenzophenone as the major product and fluorenone (FLN) and 2-nitrobenzophenone as the minor ONOO^–^-specific products. Hydrogen peroxide neither alone nor in the presence of myeloperoxidase and nitrite produces FLN or 2-nitrobenzophenone. FLN can be selectively detected using fluorescence spectroscopy, providing a chemical principle for the development of next-generation probes for ONOO^–^, with noninvasive, fluorescence-based detection of ONOO^–^-specific products. Fluorescence-based monitoring of FLN was successfully applied for the detection of ONOO^–^ generated *in situ* from the decomposition of SIN-1, a thermal source of the superoxide radical anion and nitric oxide.

## Introduction

Peroxynitrite [ONOO^–^, or oxoperoxonitrate (1-) in IUPAC nomenclature] is a reactive nitrogen species formed in biological systems in the rapid, diffusion-controlled reaction of nitric oxide (^•^NO) with the superoxide radical anion (O_2_^•–^).^1,2^ Once formed, ONOO^–^ may result in modification of biomolecules, including posttranslational modification (e.g., oxidation, nitration) of proteins^3^ and oxidation/nitration of DNA bases,^4−6^ fatty acids, and lipids.^7−9^ Formation of ONOO^–^ has been implicated in numerous diseases, typically carrying an inflammatory component, including neurodegenerative diseases, cardiovascular diseases, and cancer.^10−14^ Recently, ONOO^–^ has been shown to lead to altered recognition of cancer cells by host immune cells, providing a new mechanism of tumor immunosuppression.^15,16^

Due to its short half-life time at physiological pH and rapid reaction with carbon dioxide (CO_2_) and cellular thiols (nonenzymatic and enzymatic), detection of ONOO^–^ is typically based on the use of chemical probes and/or on detection of biomarkers of the nitrative environment.^17−19^ Nitrated tyrosine residues in proteins have been extensively used as biomarkers of ONOO^–^ production in biological systems.^20^ However, tyrosine does not react directly with ONOO^–^ but rather undergoes nitration via a multistep process involving ONOO^–^ decomposition products: nitrogen dioxide radical (^•^NO_2_), hydroxyl radical (^•^OH), and/or carbonate radical anion (CO_3_^•–^). Therefore, tyrosine nitration also occurs via ONOO^–^-independent pathways, e.g., via myeloperoxidase (MPO)-catalyzed oxidation of nitrite to ^•^NO_2_. This ambiguity about the identity of the species detected also applies to many fluorogenic chemical probes used for ONOO^–^ detection, including dihydrorhodamine (DHR) and dihydrodichlorofluorescein (DCFH), as these respond to free radicals formed from ONOO^–^ decomposition rather than ONOO^–^*per se*.^21,22^ Development of selective chemical probes for ONOO^–^ remains an area of intense research.^23−25^ Despite the numerous claimed selectivities of the reported probes for ONOO^–^, meticulous inspection of the presented data and reaction conditions typically points to the lack of absolute selectivity and/or inappropriate experimental conditions, as discussed elsewhere.^24,26^

Although boronate probes were initially developed and successfully applied for the biorthogonal detection of hydrogen peroxide (H_2_O_2_),^27−29^ they proved to serve as invaluable chemical tools for the detection and quantification of the fluxes of ONOO^–^ in cell-free and cellular systems.^26,30−32^ The major oxidation product, a corresponding phenol, is common for various nucleophilic two-electron oxidants (Scheme S1). Generation of the phenolic product results from the heterolytic cleavage of the peroxide or oxygen–halogen bond in the adduct to eliminate the anionic leaving group, accompanied by formation of the phenoxyboronate. However, the reaction between boronates and ONOO^–^ also involves a minor pathway, involving a homolytic cleavage of the peroxide bond in the adduct, resulting in the formation of ^•^NO_2_ and phenyl-type radicals and subsequently their recombination and/or reduction products.^33^ This chemical reactivity, specific for ONOO^–^, has been utilized for unambiguous identification of ONOO^–^ as the oxidant of the boronate probes in cell-free and cellular systems.^30,34−43^*ortho*-MitoPhB(OH)_2_ serves as an example of a mitochondria-targeted phenyl boronate probe that reacts with ONOO^–^ to generate a major phenolic product (*ortho*-MitoPhOH) and a minor nitrated product (*ortho*-MitoPhNO_2_).^34,44^ Recent studies have shown that in the case of *ortho*-MitoPhB(OH)_2_, the phenyl radical (*ortho*-MitoPh^•^) also undergoes rapid intramolecular cyclization, leading to the formation of the ONOO^–^-specific product *cyclo*-*ortho*-MitoPh (9,10-dihydro-9,9-diphenyl-9-phosphoniaphenanthrene).^45−47^ This minor product is unique to the ONOO^–^ reaction and is not formed by any other oxidant. However, detection and quantification of such products require chromatographic separation and are therefore of destructive character, limiting the opportunity for fluorescence-based specific imaging and real-time monitoring of ONOO^–^ and making the analysis cumbersome.

**Scheme 1:**
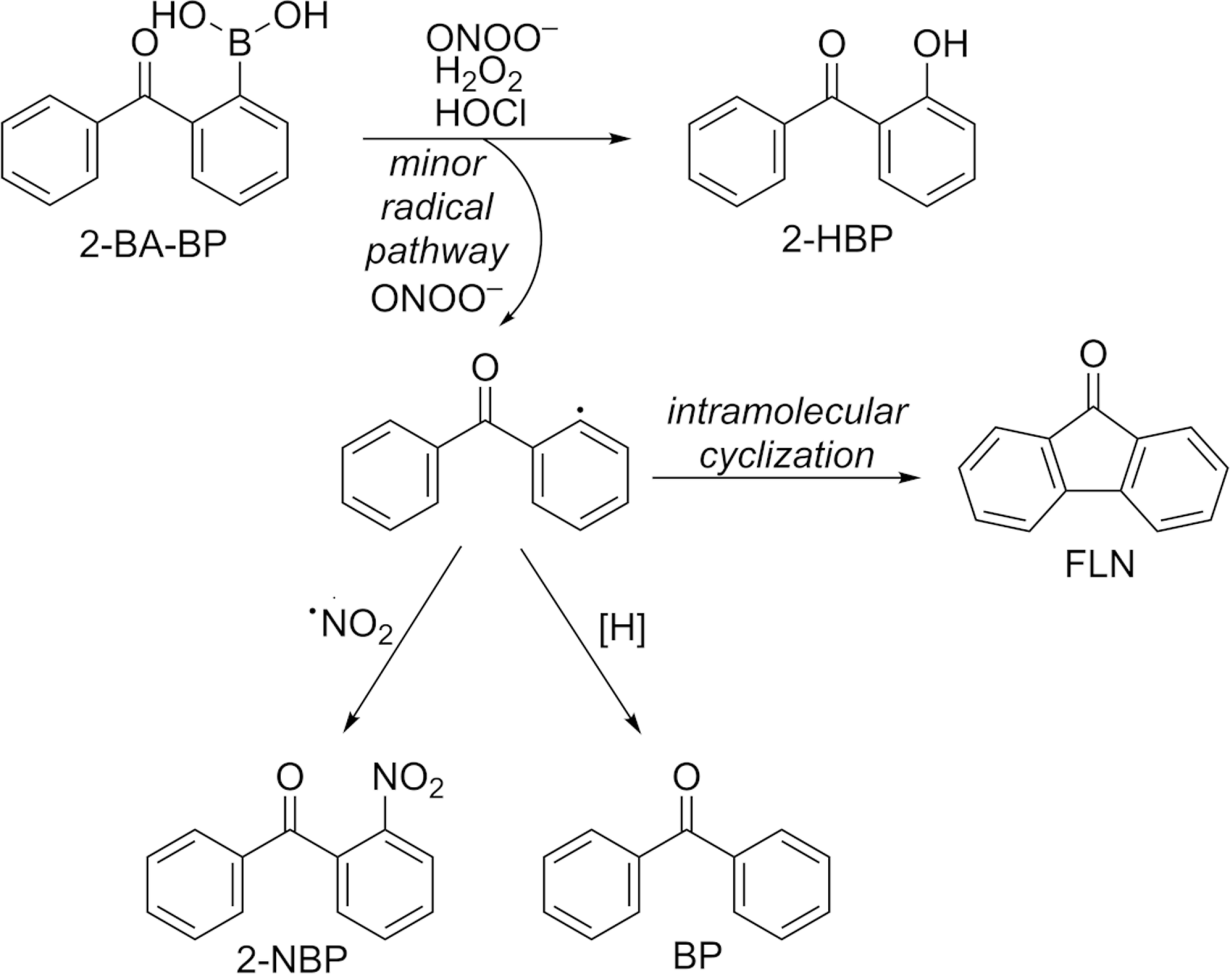
Oxidative Conversion of 2-BA-BP to the Products Identified in This Study

Here, we propose a new boronate probe design with the goal of fluorescence-based detection of ONOO^–^-specific product(s). We took advantage of the recently observed intramolecular cyclization in the reaction between *ortho*-Mito-PhB(OH)_2_ and ONOO^–^^34,44−46^ and previous reports demonstrating the formation of fluorenone from the 2-benzoylphenyl radical.^48−54^ We hypothesized that intramolecular cyclization is a general feature for various phenylboronates with an *ortho* substituent carrying phenyl radical-reactive aromatic ring(s) and that such cyclization may be used to design a chemical probe generating a fluorescent product only in the presence of ONOO^–^. As a proof of concept, we tested the reactivity of benzophenone-2-boronic acid (2-BP-BA) toward ONOO^–^ and demonstrated the formation of fluorescent 9-fluorenone (FLN) as the ONOO^–^–specific product (Scheme 1).

## Experimental Section

### Materials

All reagents and solvents were purchased from commercial vendors and used without further purification.

### Synthesis

Phenyl(2-(4,4,5,5-tetramethyl-1,3,2-dioxaborolan-2-yl)phenyl)methanone (2-BE-BP) and phenyl(4-(4,4,5,5-tetramethyl-1,3,2-dioxaborolan-2-yl)phenyl)methanone (4-BE-BP) were prepared from bromobenzophenones and bis(pinacolato)diboron using palladium–based catalysts according to the published procedure^55^ with modifications. The detailed description of synthetic procedures and corresponding ^1^H nuclear magnetic resonance spectroscopy spectra and electrospray ionization mass spectra can be found in the Supporting Information.

### High-Performance Liquid Chromatography Analysis

2-BA-BP, 4-BA-BP, and their oxidation products were separated on a Shimadzu Nexera high-performance liquid chromatography (HPLC) instrument (Kyoto, Japan) equipped with a UV–vis absorption detector.

### Kinetic Studies

The rate constants for the reaction of 2-BA-BP and 4-BA-BP with ONOO^–^ were determined using the competition kinetics methodology combined with HPLC-based detection of the reaction products. CBA was used as a reference compound, utilizing the reported rate constants of 1.1 × 10^6^ M^–1^ s^–1^.^32^ The determination of the rate constants of 2-BA-BP and 4-BA-BP with H_2_O_2_ was carried out under pseudo-first-order conditions using an excess of the oxidant. The kinetic traces were recorded at 340 or 295 nm in the case of 2-BA-BP and 4-BA-BP, respectively.

### Inhibition of ABTS^•+^ Formation

The one-electron oxidation of ABTS [2,2′-azino-bis(3-ethylbenzothiazoline-6-sulfonic acid)] in the presence of ONOO^–^ and boronates was measured spectrophotometrically at 735 nm^56^

### EPR Spin-Trapping Experiments

The spin-trapping experiments were conducted utilizing 2-methyl-2-nitrosopropane (MNP) as a spin trap using a Bruker Magnettech ESR 5000 spectrometer.

### Solid Phase Extraction

For solvent exchange from aqueous to acetonitrile, a solid phase extraction (SPE) cartridge (Thermo Fisher, SOLA HRP, 10 mg/mL) was first washed with 1 mL of MeCN, followed by 1 mL of water. The reaction mixture (0.8 mL) was applied to the SPE column, which was then washed with 1 mL of water. The columns were dried in a vacuum for 5 min, followed by 30 min under air at room temperature, and again in a vacuum for 15 min. Then, the oxidation products were eluted with 0.8 mL of dry MeCN, collected, and analyzed by HPLC and fluorimetry.

A detailed description of all experimental procedures used in this study is provided in the Supporting Information.

## Results and Discussion

### FLN Formation in the Reaction of 2-BA-BP with ONOO^–^

**Figure 1:**
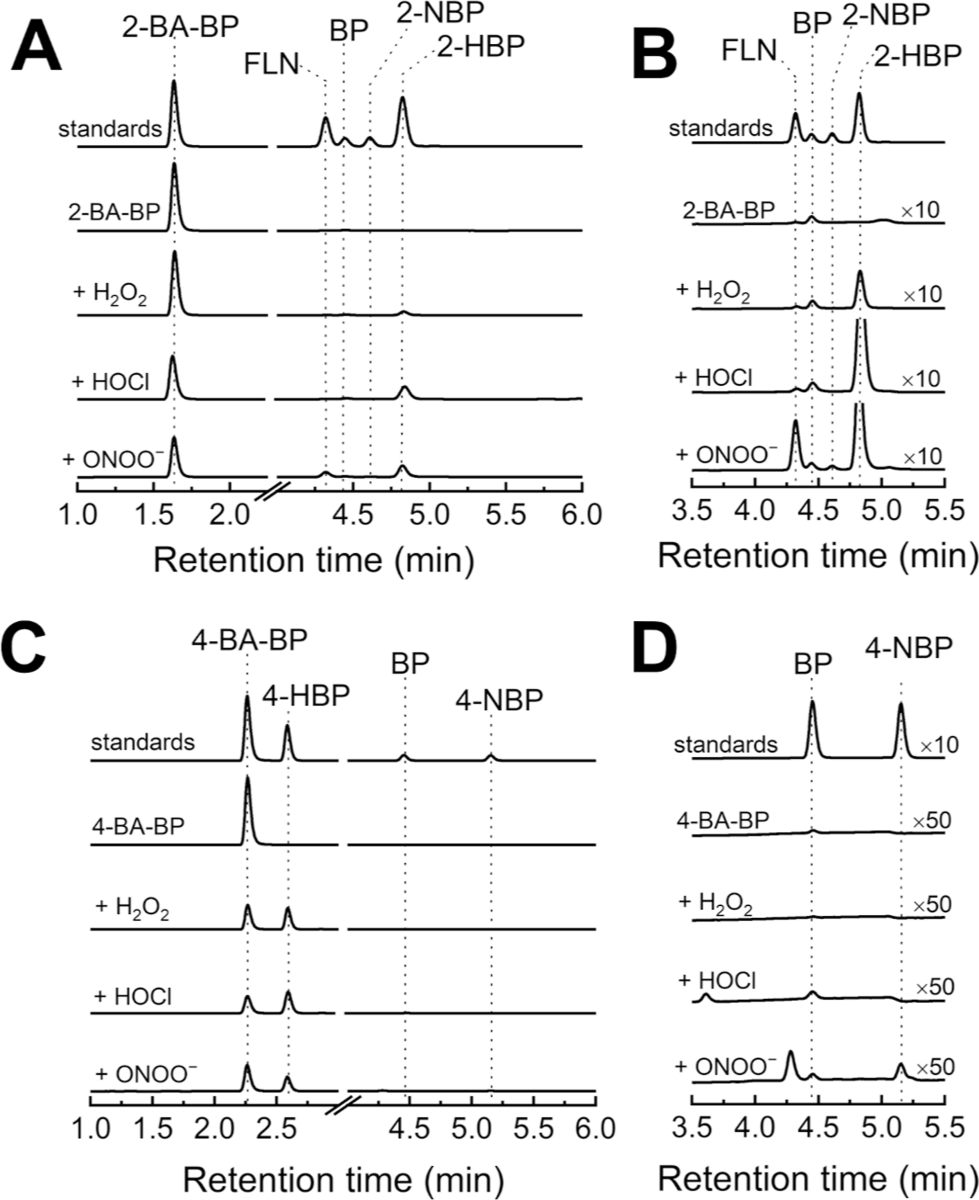
Product analyses for oxidation of 2-BA-BP and 4-BA-BP by different oxidants. HPLC chromatograms obtained after the incubation of (A,B) 2-BA-BP (100 μM) or (C,D) 4-BA-BP (100 μM) alone or in the presence of H_2_O_2_ (80 μM), HOCl (80 μM), or ONOO^–^ (80 μM) in a phosphate buffer (100 mM, pH 7.4) containing 10 μM dtpa and 1% DMSO or 1% MeCN (in experiments with HOCl). The concentration of 2-BA-BP, 2-HBP, 4-BA-BP, and 4-HBP standards was 100 μM, and the concentration of FLN, BP, 2-NBP, and 4-NBP standards was 10 μM. The probes were reacted with ONOO^–^, HOCl, or H_2_O_2_ and incubated for 5 min, 15 min, and 24 h, respectively, before HPLC analyses. Panels (B,D) show the 10× or 50× magnification of the traces shown in panel (A,C), respectively, for better visualization of the formation of minor reaction products.

To test the hypothesis that during the reaction between 2-BA-BP and ONOO^–^, the phenyl-type radical is formed and undergoes intramolecular cyclization to fluorenone, HPLC analyses were performed for the identification and quantitative analysis of the reaction products. The oxidation products of 2-BA-BP were determined by comparison with authentic standards. As shown in Figure 1A,B, in the presence of authentic ONOO^–^, 2-BA-BP is converted to 2-HBP as the major product and 2-NBP and FLN as minor products formed in the radical pathway. The formation of the nitrated product (2-NBP) is the result of a rapid recombination of 2-aroylaryl and ^•^NO_2_ radicals and is consistent with previous reports for other boronate probes. Analysis of the HPLC data revealed the buildup of another peak with retention time of 4.35 min. This retention time, as well as an online absorption spectrum, was identical to that of the authentic FLN and was assigned to FLN formed via a radical-induced intramolecular cyclization mechanism.

The specificity of the FLN product to ONOO^–^ was tested using other oxidants [H_2_O_2_ and hypochlorous acid (HOCl)], which are known to oxidize boronic compounds, and by comparing the products formed. Consistent with previous research on the chemical reactivity of boronic compounds,^30,36−43,57^ the phenolic product (2-HBP) was detected as the major product in the case of all three oxidants. In addition to 2-HBP, the products derived from the 2-aroylaryl radical (i.e., FLN and 2-NBP) were formed only during the reaction of 2-BA-BP with ONOO^–^ but not in the case of H_2_O_2_ and HOCl oxidants. Moreover, we investigated the reaction of the 2-BA-BP probe with other reactive oxygen species, including superoxide (O_2_^•–^), singlet oxygen (^1^O_2_), and a hydroxyl radical (^•^OH). As shown in Figures S1 and S2, these oxidants do not produce FLN, further supporting the specificity of this product for ONOO^–^.

To further decipher the reaction mechanism, we synthesized 4-BA-BP, an isomer of 2-BA-BP, and compared the reaction product profiles between the two isomeric benzophenone (BP) boronic acids (Figure 1C,D). The oxidation of 4-BA-BP by ONOO^–^ leads to the formation of the corresponding phenol (4-HBP, the major product) and ONOO^–^-specific products: 4-NBP and BP. However, formation of the cyclic product FLN from 4-BA-BP does not occur. With an increase in the ONOO^–^ concentration, especially at or above the concentration of the 4-BA-BP probe, another peak eluting at 4.30 min appeared in HPLC traces. This was attributed to the product of nitration of the 4-HBP phenolic product by the ^•^NO_2_ radical formed in the minor pathway and/or during decomposition of excess ONOO^–^. This was confirmed by the detection of the same product in the reaction mixture obtained when 4-HBP was mixed with ONOO^–^ (Figure S3).

**Figure 2:**
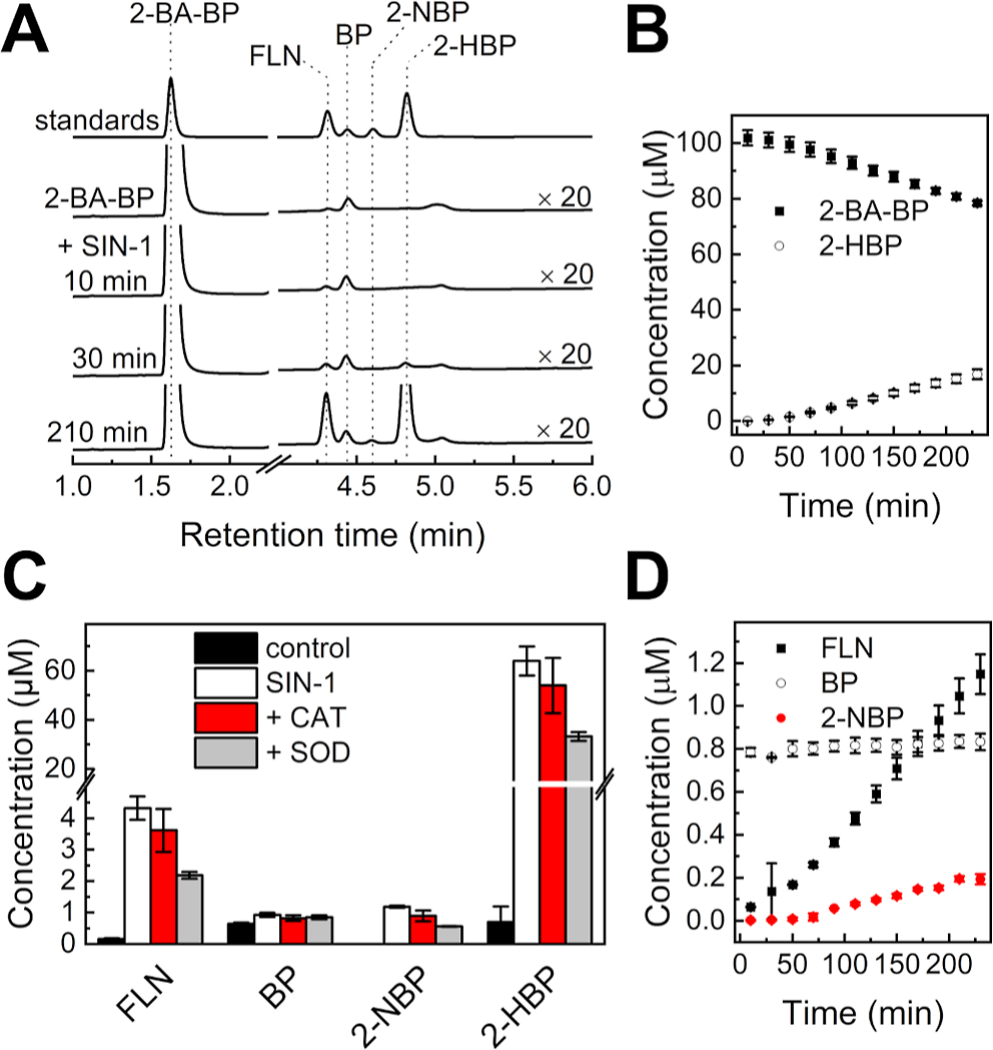
Product analyses for oxidation of 2-BA-BP by in situ generated ONOO^–^. (A) The chromatograms recorded during HPLC analyses of products formed during 2-BA-BP oxidation by *in situ* generated ONOO^–^ from SIN-1 decomposition. The concentration of 2-BA-BP and 2-HBP standards was 100 μM, and the concentration of FLN, BP, and 2-NBP standards was 10 μM. (B,D) Time dependence of substrate depletion and major (B)/minor (D) product formation during SIN-1-induced oxidation of 2-BA-BP. Incubation mixtures consisted of 100 μM 2-BA-BP and 100 μM of SIN-1 in a phosphate buffer (100 mM, pH 7.4) containing dtpa (10 μM) and DMSO (1%). (C) Concentrations of the products formed during oxidation of 2-BA-BP (100 μM) by ONOO^–^ generated from the decomposition of SIN-1 (250 μM) in the presence or absence of CAT (100 U/mL) or SOD (0.02 mg/mL) in a phosphate buffer (100 mM, pH 7.4) containing dtpa (10 μM) and DMSO (1%). The HPLC traces were collected after 4 h incubation of 2-BA-BP with SIN-1 in the presence and absence of inhibitors using the absorption detector set at 254 nm.

In order to determine the efficiency of 2-BA-BP as a probe for the detection of ONOO^–^ generated *in situ*, the oxidative conversion of 2-BA-BP was also investigated in the presence of 3-morpholinosydnonimine hydrochloride (SIN-1), cogenerating equimolar fluxes of O_2_^•–^ and ^•^NO.^58−60^ As shown in Figure 2, HPLC analyses of the incubation mixtures indicated the formation of both the major product, 2-HBP, and the minor products, 2-NBP and FLN, in a time-dependent manner over the course of 4 h incubation. Formation of those products was partially inhibitable by superoxide dismutase (SOD) but not by catalase (CAT), consistent with ONOO^–^ and not H_2_O_2_ serving as the oxidant. The lack of a complete inhibition of the probe oxidation in the presence of SOD can be explained by the dynamic competition between ^•^NO and SOD for O_2_^•–^.^32^ These results confirmed our notion that ONOO^–^, whether added as a bolus or generated *in situ*, forms the specific product FLN in the presence of the 2-BA-BP probe. As expected, FLN was not formed during the incubation of 4-BA-BP with SIN-1 (Figure S4).

### Selectivity and Product Specificity

**Figure 3:**
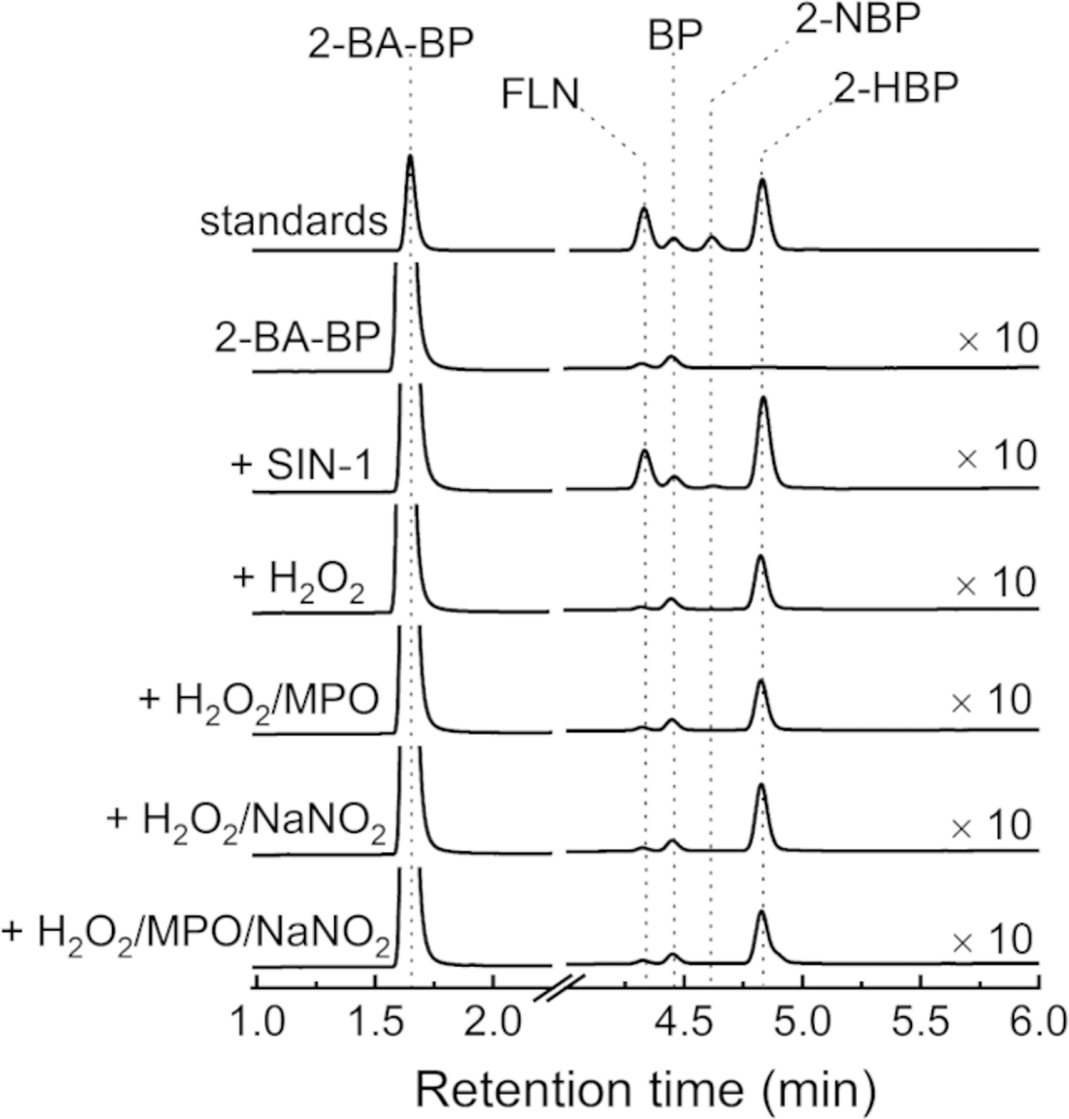
Product analyses for oxidation of 2-BA-BP in the presence of different nitrating systems. HPLC traces (254 nm) of the products detected during the reaction between the 2-BA-BP probe (100 μM) and SIN-1 (200 μM), H_2_O_2_ (1 mM), H_2_O_2_ (1 mM) + MPO (10 nM), H_2_O_2_ (1 mM) + NaNO_2_ (5 mM), and H_2_O_2_ (1 mM) + NaNO_2_ (5 mM) + MPO (10 nM) in a phosphate buffer (100 mM, pH 7.4) with dtpa (10 μM) and DMSO (1%). The products were determined 1 h after adding H_2_O_2_. The concentration of 2-BA-BP and 2-HBP standards was 100 μM, and the concentration of FLN, BP, and 2-NBP standards was 10 μM.

The most commonly used biomarkers of ONOO^–^, nitrated tyrosyl residues on proteins, are also formed on ONOO^–^-independent pathway(s), including peroxidase-catalyzed nitration in the presence of H_2_O_2_ and NO_2_^–^. To investigate whether 2-BA-BP can differentiate between ONOO^–^ and MPO/H_2_O_2_/NO_2_^–^ systems generating ^•^NO_2_, the reaction mixtures of 2-BA-BP with both nitrating reagents were analyzed by HPLC (Figure 3). Although the major product, 2-HBP, was formed in all of the systems tested, due to the presence of either ONOO^–^ or H_2_O_2_, this experiment clearly identified FLN and 2-NBP as specific products for ONOO^–^. The formation of ONOO^–^-specific minor products occurred in the presence of ONOO^–^ but not in the presence of MPO/H_2_O_2_/NO_2_^–^, consistent with the data reported previously for several other boronates, including the *ortho*-MitoPhB(OH)_2_ probe^34^ and cationic boronate probe based on a coumarin-imidazolium scaffold (CI-Bz-BA).^38^

Oxidant-dependent reactions are often altered in the presence of bicarbonate (HCO_3_^–^) (present in a dynamic equilibrium with CO_2_), ubiquitous in respiring cells. The effect of the presence of CO_2_ on boronate oxidation is oxidant-dependent. It has been reported that, in the presence of bicarbonate, H_2_O_2_ reacts with coumarin boronic acid approximately 100 times faster than H_2_O_2_ alone.^61^ This is due to the formation of a more potent oxidant, peroxymonocarbonate (HCO_4_^–^), by the reaction of CO_2_ with H_2_O_2_.^62^ Therefore, we tested whether the H_2_O_2_-derived HCO_4_^–^ reacts with 2-BA-BP and 4-BA-BP faster than H_2_O_2_ and whether additional, radical-mediated products including FLN are formed. While the rate of oxidation of the 2-BA-BP and 4-BA-BP probes in the presence of H_2_O_2_ is increased in the presence of bicarbonate in a concentration-dependent manner (Figure S5C,D), the corresponding phenols remained the sole reaction products, with no formation of FLN (Figure S5A,B). This effect is consistent with the data reported previously for the *ortho*-MitoPhB(OH)_2_ probe.^46^*Per contra*, it has been shown that CO_2_ decreases the extent of the oxidation of boronates by ONOO^–^ due to a competition between the boronate probe and CO_2_ for ONOO^–^ and a rapid decomposition of the nitrosoperoxycarbonate adduct (ONOOCO_2_^–^) formed from the reaction between ONOO^–^ and CO_2_.^30^ As shown in Figure S6A, CO_2_ dose-dependently inhibits the formation of both the major phenolic product and the minor, ONOO^–^-specific products formed in the reaction between 2-BA-BP and ONOO^–^. This is consistent with the ONOO^–^ scavenging effect of CO_2_ and suggests the lack of the formation of a phenyl-type radical from the interaction of 2-BA-BP with ONOOCO_2_^–^-derived radicals.

Glutathione (GSH) is a biological reductant present at millimolar concentrations in living cells and reacts directly with ONOO^–^ with a rate constant of ∼ 1.3 × 10^3^ M^–1^ s^–1^ (at 37 °C and pH 7.5).^31,37^ GSH may also interact with and reduce the phenyl-type radical, lowering the yield of FLN. Therefore, we investigated the influence of GSH on the profiles of major and minor oxidation products (Figure S6B). The presence of 0.5–7.5 mM GSH during the oxidation of 2-BA-BP by ONOO^–^ led to the inhibition of 2-HBP, FLN, and 2-NBP formation, whereas a small increase in the BP concentration was observed. However, even in the presence of 7.5 mM GSH, ONOO^–^ reacted with 2-BA-BP, forming 2-HBP, FLN, and 2-NBP. The partial reduction of the major and minor, ONOO^–^-specific product formation can be explained in terms of the competition between 2-BA-BP and GSH for ONOO^–^. The observed increase in BP formation is attributed to GSH donating a hydrogen atom to the phenyl radical. Overall, the results obtained indicate that even in the presence of physiological concentrations of GSH and HCO_3_^–^, the 2-BA-BP probe may still intercept a fraction of ONOO^–^ and produce the corresponding phenol, 2-HBP, as well as the ONOO^–^-specific products, FLN and 2-NBP.

### Fluorescence Measurements

**Figure 4:**
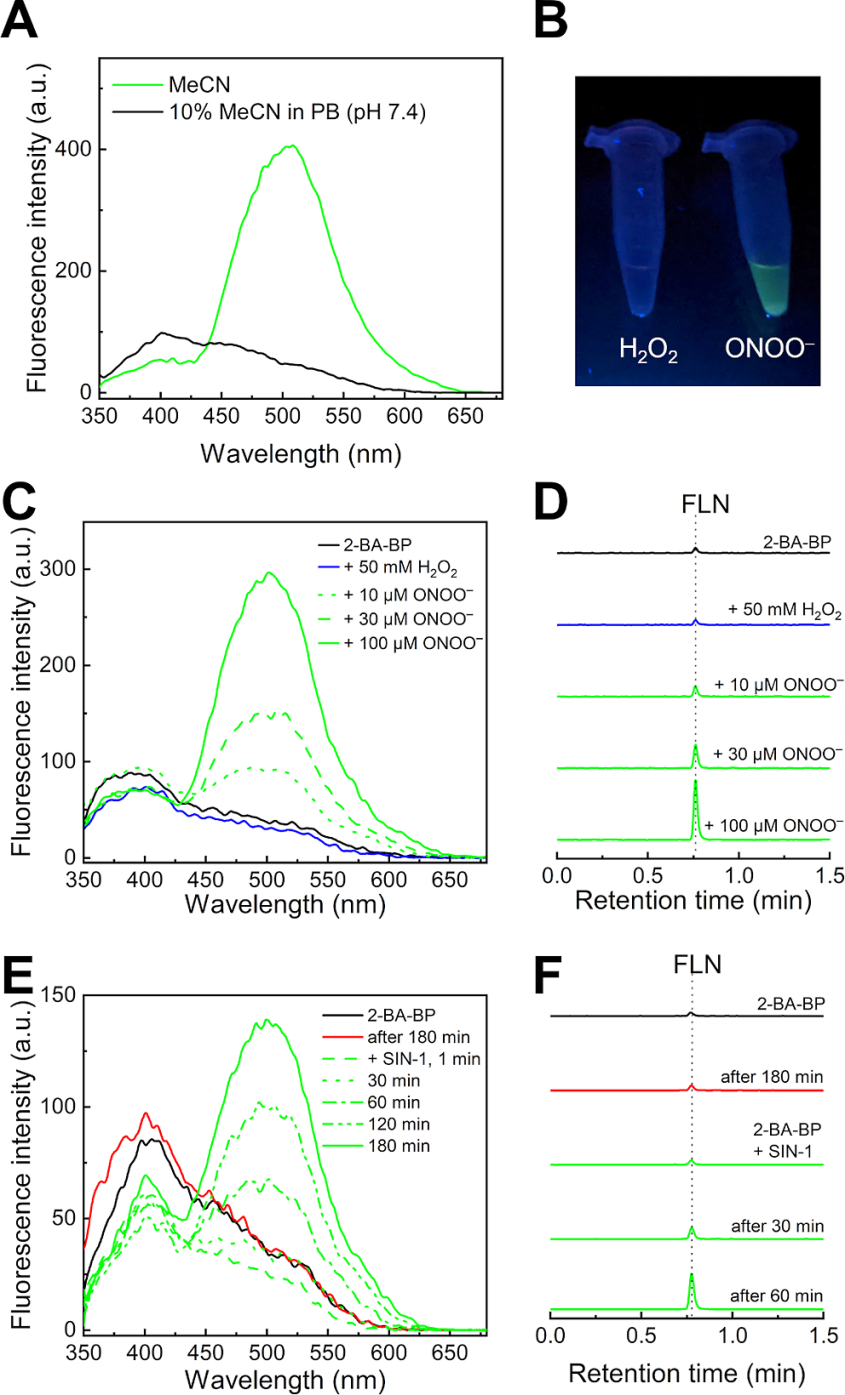
Fluorescence-based analyses of the reaction mixtures of 2-BA-BP with H_2_O_2_, ONOO^–^, and SIN-1. (A) Fluorescence emission spectra of FLN (1 μM) in MeCN and in aqueous solution containing phosphate buffer (0.1 M, pH 7.4), MeCN (10%), and dtpa (10 μM). λ_ex_ = 248 nm, ex/em slits: 10/20 nm. (B) Images showing the fluorescence of the SPE extracts upon ultraviolet light exposure. 2-BA-BP (100 μM) was reacted with ONOO^–^ (100 μM) or H_2_O_2_ (20 mM), and the products were extracted and concentrated (25×) to acetonitrile using SPE. (C) Fluorescence emission spectra of the extracts. 2-BA-BP (100 μM) was reacted with ONOO^–^ (0–100 μM) or H_2_O_2_ (50 mM) in aqueous solution containing phosphate buffer (0.1 M, pH 7.4), and the products were extracted to acetonitrile using SPE. (D) Rapid HPLC analyses of the FLN product in the extracts. The traces were collected using the fluorescence detector set at λ_ex_ = 256 nm, λ_em_ = 505 nm. (E,F) Specific fluorescence-based detection of ONOO^–^ produced during SIN-1 decomposition. 2-BA-BP (100 μM) was incubated with SIN-1 (100 μM) in aqueous solution containing phosphate buffer (0.1 M, pH 7.4), and the products were extracted to acetonitrile using SPE.

While simple derivatives of benzophenone exhibit weak fluorescence, FLN displays an absorption band in the ultraviolet region and strong green fluorescence with a maximum located at 500 nm (Figure S7). However, it is observed only in organic solvent (e.g., acetonitrile, dichloromethane, ethyl acetate, tetrahydrofuran, and hexane).^63−65^ The replacement of a pure acetonitrile solvent by an aqueous solution containing phosphate buffer and 10% acetonitrile (MeCN) results in strong suppression of the fluorescence signal of FLN (Figure 4A). This significantly limits the applicability of 2-BA-BP for the nondestructive, real-time fluorescence detection of ONOO^–^ in an aqueous environment. To overcome this limitation, fluorescence-based analysis of the reaction mixtures of 2-BA-BP with ONOO^–^ was carried out after the solvent was exchanged from aqueous phosphate buffer to acetonitrile using an SPE approach (Figure 4B–D). Upon solvent exchange, the fluorescence signal due to FLN formation could be detected, and it revealed increased production of FLN upon the addition of bolus ONOO^–^ but not H_2_O_2_. This approach was subsequently applied to fluorescence-based monitoring of ONOO^–^ produced *in situ* from superoxide and nitric oxide fluxes generated upon SIN-1 decomposition (Figure 4E). In the case of both bolus and SIN-1-derived ONOO^–^, rapid HPLC analyses of the extracts confirmed the identity of the fluorescent product as FLN (Figure 4D,F).

### Stoichiometric Analysis

To determine the reaction stoichiometry, 2-BA-BP and 4-BA-BP were incubated with different concentrations of ONOO^–^ and H_2_O_2_, and the reaction mixtures were analyzed by HPLC. The reactions between 2-BA-BP or 4-BA-BP and ONOO^–^ show close to 1:1 stoichiometry, consistent with previous results for simple arylboronates (Figure S8).^26,30^ A small deviation in the stoichiometry, especially for 2-BA-BP, may be due to the presence of organic cosolvents in the reaction mixture. In the case of H_2_O_2_, HPLC analyses indicate a low yield of 2-HBP under the experimental conditions used (Figure S9A). This can be attributed to the low value of the rate constant of the reaction (see below), which may significantly limit the applicability of the probe to detect H_2_O_2_ in biological systems. In contrast with 2-BA-BP, the reaction between 4-BA-BP and H_2_O_2_ shows 1:1 stoichiometry, in the case of both probe consumption and product formation, with respect to the amount of the oxidant added (Figure S9B).

### Reaction Kinetics

To determine the rate constant of the reaction between 2-BA-BP or 4-BA-BP and ONOO^–^, the competition kinetics approach was used, with the coumarin-7-boronic acid (CBA) probe serving as the competitor (Figure S10). The value of 1.5 × 10^5^ M^–1^s^–1^ obtained for 2-BA-BP is significantly lower than the values reported for most boronate-based probes.^26,30,35−39,43,66^ On the other hand, the 4-BA-BP isomer reacts with ONOO^–^ with the rate constant close to a value of 1 × 10^6^ M^–1^ s^–1^, more typical for boronic probes. The rate constant of the reaction of ONOO^–^ with the 2-BA-BP probe is almost 7-fold lower, which we tentatively attribute to intramolecular interaction between the carbonyl and the boronic acid moieties in 2-BA-BP.^67,68^ The rate constant for the reaction of 2-BA-BP and H_2_O_2_ was also determined, and the value of (1.4 ± 0.4) × 10^–1^ M^–1^ s^–1^ was obtained (Figure S11A). In the case of the 4-BA-BP isomer, the rate constant for the reaction with H_2_O_2_ of *k* = 2.4 ± 0.1 M^–1^ s^–1^ was determined (Figure S11B), and it is close to the values reported for other boronate-based redox probes.^26,30,35−39,43,66^ All of these data suggest decreased reactivity of the 2-BA-BP toward nucleophilic oxidants, which could be rationalized by a direct interaction of the boronic acid moiety located in the *ortho* position to the carbonyl group.

### Effect of Phenyl Radical Scavengers

To further demonstrate the intermediacy of the phenyl-type radical in the formation of ONOO^–^-specific products, FLN and 2-NBP, we examined the effect of 2-propanol (2-PrOH), an efficient phenyl radical scavenger, on the profile of the oxidation products of 2-BA-BP upon addition of ONOO^–^ (Figure S12). The yield of FLN was significantly decreased when the reaction between 2-BA-BP and ONOO^–^ was performed in the presence of 10% 2-PrOH. The dominant minor product formed from the radical pathway, detected in the presence of 2-PrOH was BP, which is formed by a hydrogen abstraction from 2-PrOH by the 2-aroylaryl radical. The conversion of the 2-aroylaryl radical in the presence of 2-PrOH into the product in which the boronate moiety is replaced by a hydrogen atom explains a smaller fraction undergoing intramolecular cyclization to FLN and supports the mechanism involving the 2-aroylaryl radical in FLN formation during the reaction between 2-BA-BP and ONOO^–^.

### ABTS Oxidation

Based on the previous results observed for the *ortho*-MitoPhB(OH)_2_ probe,^34^ we reasoned that intramolecular cyclization of the phenyl-type radical should significantly decrease the yield of the oxidizing radicals formed from ONOO^–^. To this end, a colorimetric probe for one-electron oxidants, ABTS, was incubated with ONOO^–^ in the presence and absence of the probes, and the formation of the ABTS radical cation was monitored by spectrophotometry. The addition of ONOO^–^ to a solution of ABTS led to the formation of ABTS^•+^, which is reflected in the appearance of the absorption band with a maximum at 735 nm. As shown in Figure S13, the extent of oxidation of ABTS by ONOO^–^-derived radicals was decreased in the presence of 2-BA-BP and 4-BA-BP and was almost completely suppressed by *ortho*-MitoPhB(OH)_2_. Because 2-BA-BP and *ortho*-MitoPhB(OH)_2_ reduce the oxidative activity of ONOO^–^ to a greater extent than 4-BA-BP, we conclude that the intramolecular cyclization process is responsible for the reduced extent of ABTS oxidation.

### EPR Spin Trapping

The electron paramagnetic resonance (EPR) study using a 2-methyl-2-nitrosopropane (MNP) spin trap was performed to detect and characterize radical intermediates formed during the reaction between ONOO^–^ and isomers of benzophenone boronic acid (Figure S14). The addition of a bolus amount of ONOO^–^ to incubations containing 2-BA-BP or 4-BA-BP and MNP resulted in the appearance of the EPR spectra that can be attributed to the spin adducts of the benzophenone-derived phenyl radical. In the case of the 2-BA-BP probe, the signal intensity was significantly lower than that detected for the 4-BA-BP isomer. This further confirms a rapid intramolecular recombination mechanism of the 2-aroylaryl radical generated in the reaction of the 2-BA-BP probe with ONOO^–^. The signal intensity of the MNP-phenyl radical adducts decreased in the presence of 2-PrOH, corroborating the direct scavenging of the 2-BA-BP- and 4-BA-BP-derived phenyl radicals by 2-PrOH.

### Limitations and Future Directions

Here, we report the design of a boronate-based probe that produces a fluorescent product in the ONOO^–^-specific pathway for fluorescence-based selective monitoring of ONOO^–^ formation (Scheme 1). Phenyl-type radical intermediate undergoes intramolecular cyclization to form a fluorescent product. However, in the case of the 2-BA-BP probe, the proposed methodology requires solvent exchange due to low fluorescence yield of FLN in aqueous solutions. Moreover, the reaction rate constant indicates lower reactivity of 2-BA-BP than that observed for other aromatic boronates, which can be attributed to the interaction of the boronic acid moiety with the carbonyl group of benzophenone. Future directions in developing a novel class of fluorescent probes for rigorous detection and quantitative analyses of ONOO^–^ in biological systems, based on formation of the ONOO^–^-specific product via a radical-induced intramolecular cyclization mechanism, should include the improvement of the rate of reaction and application of water-compatible fluorophores.

## Conclusions

While boronates are widely used in redox biology research to detect cellular oxidants, there is a continuous discussion and uncertainty about the identity of the actual oxidant(s) being reported. In most cases, H_2_O_2_ or ONOO^–^ are claimed to be the oxidants involved. Currently, pharmacological treatments, such as specific inhibitors and scavengers, are required to determine the oxidant responsible for the fluorescence signal. Here, we overcome this limitation by providing a new approach for specific detection of ONOO^–^, based on fluorogenic cyclization of the phenyl type-radical formed during oxidation of a boronate probe by ONOO^–^, with the production of a fluorescent product. Fluorenone can be selectively detected using fluorescence spectroscopy; the reported reaction provides a model and chemical principles for the development of next-generation probes for the noninvasive, fluorescence-based specific detection of ONOO^–^. The proposed design represents a proof of concept for fluorogenic cyclization of the phenyl-type radical and provides a basis for rational design of boronate-based probes producing easily detectable, peroxynitrite-specific product(s).

## Supporting Information Available

Detailed synthesis and experimental procedures; nuclear magnetic resonance spectroscopy and mass spectrometry spectra; additional experimental data: absorption and emission spectra of FLN; analyses of FLN formation from 2-BA-BP in the presence of selected oxidants; oxidation of the 4-BA-BP probe by ONOO^–^; HPLC analyses of products formed during oxidation of 4-BA-BP by *in situ* generated ONOO^–^ from SIN-1; the effect of NaHCO_3_ on 2-BA-BP and 4-BA-BP oxidation by H_2_O_2_; the effect of NaHCO_3_ and GSH on 2-BA-BP oxidation by ONOO^–^; HPLC-based titration of 2-BA-BP and 4-BA-BP with ONOO^–^ and H_2_O_2_; kinetics of the reaction of 2-BA-BP and 4-BA-BP with ONOO^–^ and H_2_O_2_; effect of 2-PrOH on products formed during the reaction between 2-BA-BP and ONOO^–^; inhibition of ONOO^–^-induced ABTS oxidation by tested probes; and EPR spin trapping of the radical intermediates. The Supporting Information is available free of charge at https://pubs.acs.org/doi/10.1021/acs.analchem.4c06983.
