# Supplementary material for: Phenyl Radical-Mediated Fluorogenic Cyclization for Specific Detection of Peroxynitrite
Source: Anal Chem. 2025 Mar 27;97(13):7299–306. doi: 10.1021/acs.analchem.4c06983 (PMC11983361; doi:10.1021/acs.analchem.4c06983)
Supplement: Supplementary file 1 — ac4c06983_si_001.pdf [file ac4c06983_si_001.pdf]

Supporting Information

Phenyl radical-mediated fluorogenic cyclization for specific detection of peroxynitrite

Aleksandra Grzelakowska[1,2,*], Radosław Podsiadły[1], Jacek Zielonka[2,*]

[1]Institute of Polymer and Dye Technology, Faculty of Chemistry, Lodz University of Technology, Stefanowskiego 16, 90-537 Lodz, Poland

[2]Department of Biophysics, Medical College of Wisconsin, 8701 Watertown Plank Road, Milwaukee, WI 53226, United States

[*]Corresponding authors (emails: aleksandra.grzelakowska@p.lodz.pl; jzielonk@mcw.edu)

Table of Contents

Scheme S1. Oxidation of arylboronates by various oxidants and mechanism of the reaction between arylboronates and $ONOO^-$ S3

Experimental Details........ S3

Figure S1. Analysis of FLN formation from 2-BA-BP in the presence of selected oxidants S8

Figure S2. Analysis of FLN formation from 2-BA-BP in the presence of hydroxyl radical S8

Figure S3. HPLC analyses of the products formed upon oxidation of the 4-BA-BP probe by $ONOO^-$. S9

Figure S4. The chromatograms recorded upon HPLC analyses of products formed during oxidation of 4-BA-BP by *in situ*-generated $ONOO^-$ from SIN-1 .... S9

Figure S5. The effect of $NaHCO_3$ on 2-BA-BP and 4-BA-BP oxidation by H_2O_2 .... S10

Figure S6. Product analyses for oxidation of 2-BA-BP in the presence of biologically relevant $ONOO^-$ scavengers S11

Figure S7. Spectroscopic characterization of FLN S11

Figure S8. Reaction stoichiometry for oxidation of 2-BA-BP and 4-BA-BP by $ONOO^-$ S12

Figure S9. HPLC-based titration of 2-BA-BP and 4-BA-BP with H_2O_2........ S13

Figure S10. Kinetics of the reaction of 2-BA-BP and 4-BA-BP with $ONOO^-$. S13

Figure S11. Kinetics of the reaction of 2-BA-BP and 4-BA-BP with H_2O_2........ S14

Figure S12. Effect of 2-PrOH, on products formed during the reaction between 2-BA-BP and $ONOO^-$. S15

Figure S13. Inhibition of $ONOO^-$–induced ABTS oxidation by tested probes........ S16

Figure S14. EPR spin trapping of the radical intermediates S16

Figure S15. ^{1}H NMR spectrum of synthesized 2-BE-BP S17

Figure S16. ^{1}H NMR spectrum of synthesized 4-BE-BP S18

Figure S17. HRMS ESI spectrum of synthesized 2-BE-BP S19

Figure S18. HRMS ESI spectrum of synthesized 4-BE-BP S20

References S21

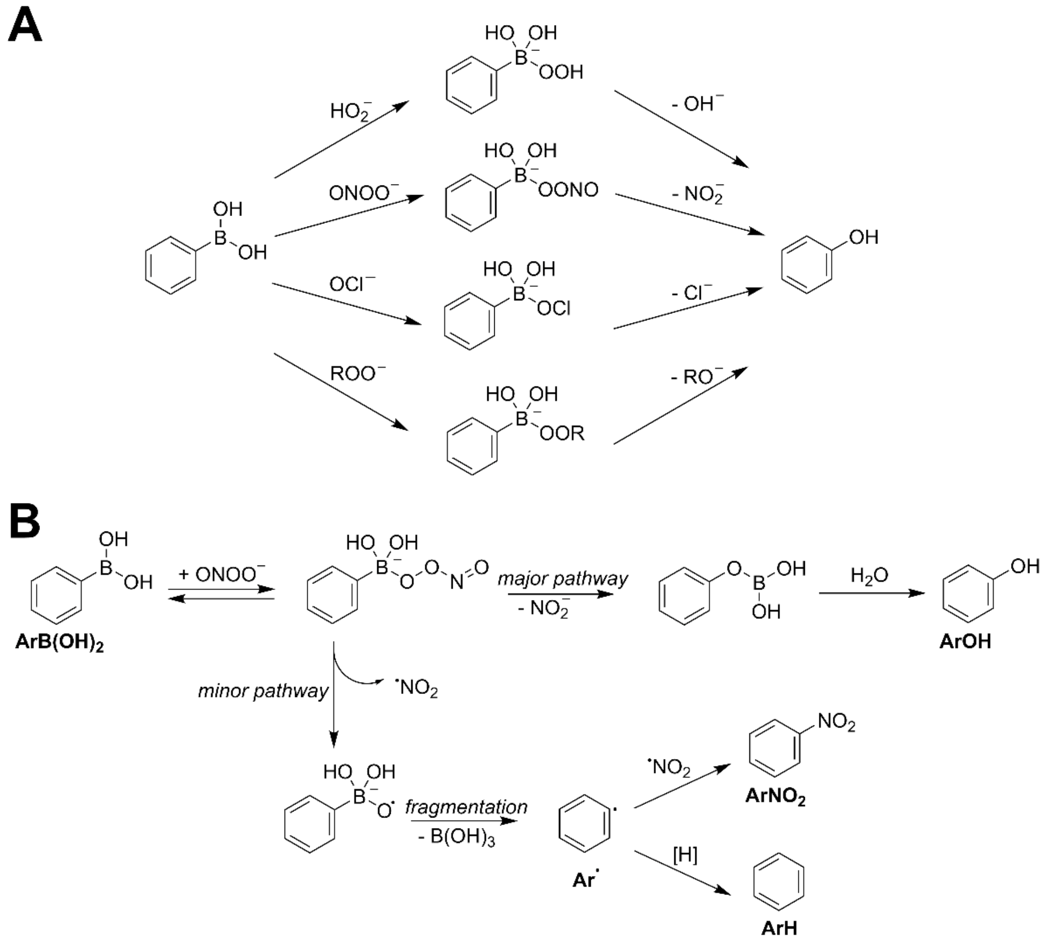

Scheme S1. (A) Oxidation of arylboronates by various oxidants, leading to a common phenolic product. (B) Mechanism of the reaction between arylboronates and $ONOO^-$ with the formation of $ONOO^-$-specific products.

Experimental Details

Materials

Benzoyl chloride, bromobenzene, benzophenone, 2-hydroxybenzophenone, 2-bromobenzophenone, 2-nitrobenzophenone, 4-hydroxybenzophenone, 4-nitrobenzophenone, and fluorenone were purchased from Sigma-Aldrich (St. Louis, MO) and were of the purest grade available. $ONOO^-$ was prepared as reported previously[1] and stored at −80 °C. MPO and SIN-1 were from Calbiochem (San Diego, CA).

Synthesis and characterization of the probe and standards of the products

Since the 2-BE-BP probe purity was 99% and we identified BP and FLN as impurities, one can conclude that the reaction of 2-bromobenzophenone and bis(pinacolato)diboron using palladium-based catalysts under microwave conditions led to the generation of 2-aroylaryl radicals. (4-bromophenyl)(phenyl)methanone was prepared from bromobenzene and benzoyl chloride through Friedel-Crafts reaction by adopting the procedure described in the literature.[2] The synthesis of coumarin-7-boronic acid (CBA) was performed based on the procedures described in the literature.[3] Nuclear magnetic resonance spectroscopy (NMR) and mass spectrometry studies confirmed the chemical structures of the compounds. Spectroscopic signals are consistent with those described in the literature.

A monomode microwave reactor (CEM Discover, Charlotte, NC) equipped with an IntelliVent pressure control system was used for the synthesis of 2-BE-BP and 4-BE-BP. Temperature of the reaction

mixtures was measured with an external infrared sensor. Analytical thin layer chromatography was performed using precoated aluminum–backed plates (Merck Kieselgel 60 F254) and visualized by ultraviolet irradiation. The purity of the compounds was determined by HPLC analyses using a Shimadzu Nexera HPLC system (Kyoto, Japan) equipped with a diode array detector. Proton nuclear magnetic resonance (^{1}H NMR) spectra were acquired on a Bruker Avance DPX spectrometer (Billerica, MA) operating at 700 MHz and were referenced according to the residual peak of the solvent based on the literature data. Spectra were visualized and analyzed using MestReNova software. Chemical shifts (δ) were quoted in ppm and coupling constants (*J*) in Hz. The abbreviations *s*, *d*, *dd*, *m* refer to the singlet, doublet, doublet of doublets, and multiplet, respectively. Solutions were prepared in deuterated chloroform ($CDCl_3$) and tetramethylsilane was used as an internal standard. High-resolution mass spectrometry (HRMS) measurements were performed using Synapt G2-Si mass spectrometer (Waters Corporation, Milford, MA) equipped with an electrospray ionization (ESI) source and quadrupole time-of-flight mass analyzer. The mass spectrometer was operated in the positive ion detection mode. The measurement was performed with capillary voltage set to 2.7 kV and sampling cone to 20 V. The source temperature was 110 °C. The results of the measurements were processed using the MassLynx 4.1 software (Waters) included with the instrument.

Detailed synthetic procedures

Synthesis of phenyl(2-(4,4,5,5-tetramethyl-1,3,2-dioxaborolan-2-yl)phenyl)methanone (2-BE-BP)

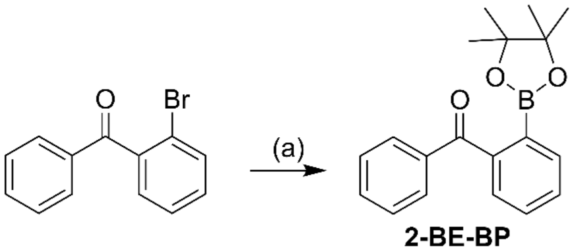

(a) B_2Pin_2, Pd(dppf)Cl_2; KOAc; 1,4-dioxane; 100 °C; MW; N_2; 45 min

(2-Bromophenyl)(phenyl)methanone (140 µL, 0.756 mmol), bis(pinacolato)diboron (232 mg, 0.950 mmol), Pd(dppf)Cl_2 (28 mg, 0.039 mmol), and potassium acetate (220 mg, 2.24 mmol) in anhydrous 1,4-dioxane (3 mL) were placed in a pressure vial equipped with a magnetic stirrer. The mixture was stirred for 5 min at room temperature, and subsequently the reaction was carried out under microwave conditions for 45 min at 100 °C under nitrogen. After that, the reaction mixture was diluted with dichloromethane (10 mL) and washed with water (2 × 10 mL). The organic layer was dried over anhydrous magnesium sulfate and evaporated under reduced pressure. The crude product was purified by column chromatography (silica gel, hexane/ethyl acetate from 20:1 to 9:1 , *v/v*) to give a white solid (58 mg, 25%).

^{1}H NMR (700 MHz, $CDCl_3$): δ 7.82-7.79 (*m*, 2H), 7.78-7.75 (*m*, 1H), 7.58-7.53 (*m*, 3H), 7.52-7.48 (*m*, 1H), 7.47-7.44 (*m*, 2H), 1.21 (*s*, 12H); HRMS (ESI): *m/z* calcd. for $C_{19}H_{21}O_3BNa$ $[M+Na]^+$: 331.1481, found: 331.1487.

Synthesis of phenyl(4-(4,4,5,5-tetramethyl-1,3,2-dioxaborolan-2-yl)phenyl)methanone (4-BE-BP)

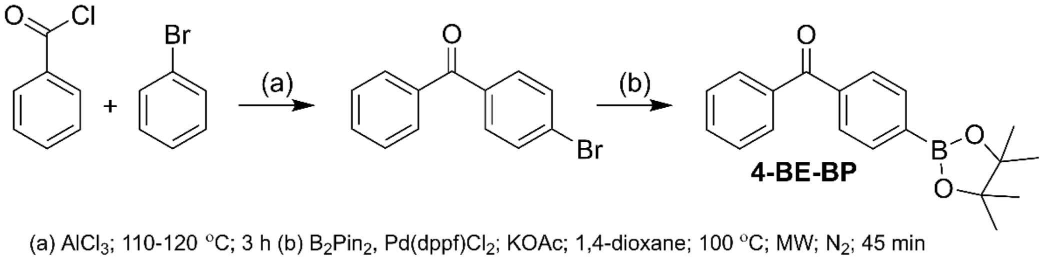

(a) $AlCl_3$; 110-120 °C; 3 h (b) B_2Pin_2, Pd(dppf)Cl_2; KOAc; 1,4-dioxane; 100 °C; MW; N_2; 45 min

Benzoyl chloride (14 mL, 0.12 mol) was added dropwise to a mixture of bromobenzene (13.2 mL, 0.1 mol) and aluminum chloride (16 g, 0.12 mol) at room temperature. Then, the mixture was refluxed for 3 h. After that, the reaction was quenched with ice cold water and extracted with chloroform (3 × 30 mL). The combined organic phase was washed with water, sodium carbonate solution, and brine; dried over magnesium sulfate; filtered; and evaporated. The crude (4-bromophenyl)(phenyl)methanone (5 g) was used directly for next step without further purification.

(4-Bromophenyl)(phenyl)methanone (200 mg, 0.756 mmol), bis(pinacolato)diboron (232 mg, 0.950 mmol), Pd(dppf)Cl_2 (28 mg, 0.039 mmol), and potassium acetate (220 mg, 2.24 mmol) in anhydrous 1,4-dioxane (3 mL) were placed in a pressure vial equipped with a magnetic stirrer. The mixture was stirred for 5 min at room temperature, and subsequently the reaction was carried out under microwave conditions for 60 min at 100 °C under nitrogen. After that, the reaction mixture was diluted with dichloromethane (10 mL) and washed with water (2 × 10 mL). The organic layer was dried over anhydrous magnesium sulfate and evaporated under reduced pressure. The crude product was purified by column chromatography (silica gel, hexane/ethyl acetate 50:1, *v/v*) to give a white solid (50 mg, 22%). ^{1}H NMR (700 MHz, $CDCl_3$): δ 7.94 (*d*, *J* = 8.0 Hz, 2H), 7.82 (*d*, *J* = 7.2 Hz, 2H), 7.79 (*d*, *J* = 8.0 Hz, 2H), 7.61 (*t*, *J* = 7.4 Hz, 1H), 7.50 (*t*, *J* = 7.7 Hz, 2H), 1.39 (s, 12H). HRMS (ESI): *m/z* calcd. for $C_{19}H_{22}O_3B$ $[M+H]^+$: 309.1662, found: 309.1662.

Preparation of probe test solutions

The stock solutions of 2-BE-BP and 4-BE-BP, and of the standards of the oxidation products (100 mM and/or 10 mM) were prepared in DMSO and stored at −20 °C. For experiments involving HOCl, both probes were dissolved in MeCN to avoid the scavenging effect of DMSO on HOCl.[4]

The concentration of $ONOO^-$ in alkaline aqueous solutions (pH > 12) was determined by measuring the absorbance at 302 nm (ε = 1.7 ×10^3 $M^{-1}cm^{-1}$). The concentrations of H_2O_2 and HOCl were also determined by spectrophotometry, using the extinction coefficient values of 39.4 $M^{-1}cm^{-1}$ (at 240 nm, in water) and 350 $M^{-1}cm^{-1}$ (at 292 nm, in 0.1 M sodium hydroxide), respectively. The working solutions of oxidants were freshly prepared before each experiment and kept on ice. Ultraviolet-visible spectrophotometry (UV-vis) absorption spectra were collected using a Jasco V–670 UV-vis–near infrared spectrophotometer (Jasco, Japan). Solutions of oxidants were added directly to the buffered solutions of the probes, and then the reaction mixtures were analyzed using the HPLC method, as described below. All measurements were performed in aqueous solutions of phosphate buffer (100 mM, pH 7.4) containing dtpa (10 or 100 μM). In reactions involving HOCl, dtpa was omitted to avoid scavenging of HOCl by dtpa.[5] To investigate the effect of CO_2 on the yield of the products of the reaction of the probes with $ONOO^-$ or H_2O_2, we carried out the reactions in the presence of sodium bicarbonate ($NaHCO_3$), with pH adjusted to 7.4.

HPLC analysis

During the analyses it was noticed that 2-BE-BP and 4-BE-BP (pinacolate esters) undergo fast hydrolysis to the boronic acid forms (2-BA-BP and 4-BA-BP) upon dilution in the aqueous phosphate buffer. Therefore, although 2-BE-BP or 4-BE-BP were added to the investigated mixtures, 2-BA-BP and 4-BA-BP were the tested species. 2-BA-BP, 4-BA-BP, and their oxidation products were separated on a Shimadzu Nexera HPLC instrument (Kyoto, Japan) equipped with UV−vis absorption detector. Typically, 20 μL of sample was injected into the HPLC system equipped with a reverse–phase column (Phenomenex, Kinetex Biphenyl, 50 mm × 4.6 mm, 2.6 μm) equilibrated with 25% MeCN [containing 0.1% (v/v) trifluoroacetic acid] in 0.1% trifluoroacetic acid aqueous solution. Gradient elution was performed at a flow rate of 1.5 mL/min. The compounds were eluted by raising MeCN concentration (v/v) from 25% to 50% over 5.5 min followed by an increase to 100% from 5.5 to 7 min. Under those conditions 2-BA-BP eluted at 1.65 min, 4-BA-BP at 2.25 min, 4-HBP at 2.60 min, FLN at 4.35 min, BP at 4.45 min, 2-NBP at 4.60 min, 2-HBP at 4.85 min, and 4-NBP at 5.15 min. The compounds were detected and quantified by monitoring the absorption at 254 ± 4 nm. The concentrations were determined based on the calibration curves obtained for authentic standards.

Kinetic studies

To a series of solutions containing 0–100 μM 2-BA-BP or 0–50 μM 4-BA-BP and a constant concentration of CBA (5 or 20 μM), $ONOO^-$ (1 or 10 μM) was added. The ratio of the two rate constants was determined from a plot of the reciprocal of the 7-hydroxycoumarin (COH) concentration versus the probe to CBA concentration ratios. The following equation was used to describe the competition and determine the rate constants:

$$\frac{1}{[COH]} = \frac{1}{[COH]_0} + \frac{1}{[COH]_0} \cdot \frac{k_{probe}}{k_{CBA}} \cdot \left(\frac{[probe]}{[CBA]}\right)$$

The determination of the rate constants of 2-BA-BP and 4-BA-BP with H_2O_2 was carried out under pseudo-first-order conditions, using an excess of the oxidant. The kinetic traces recorded at 340 nm or 295 nm, in the case of 2-BA-BP and 4-BA-BP, respectively, were fitted to a single-exponential function corresponding to the pseudo-first-order kinetics. The second-order rate constant was determined from the slopes of the plots of the observed pseudo-first-order rate constants versus the concentration of H_2O_2.

Oxidation of the probe induced by $ONOO^-$ produced from SIN-1

The HPLC analysis was used to monitor the decay of SIN-1 and 2-BA-BP, and to identify the products formed. To initiate the reaction, SIN-1 (100 or 250 μM) was added to a phosphate buffer solution (100 mM, pH 7.4) containing dtpa (10 μM) and 2-BA-BP (100 μM) in the presence or absence of CAT (100 U/mL) or SOD (0.02 mg/mL). The analysis was performed using a Shimadzu HPLC instrument equipped with an absorption detector, as described above. Changes in the peak area were used to determine the progress of the reaction over the time. Data are means ± standard deviation of three independent experiments.

Oxidation of 2-BA-BP induced by H_2O_2 in the presence of MPO and $NaNO_2$

2-BA-BP (100 μM) was incubated with MPO (10 nM), H_2O_2 (1 mM), and $NaNO_2$ (5 mM) in a phosphate buffer (100 mM, pH 7.4) containing dtpa (10 μM) at room temperature for 60 min. Reactions were terminated by adding CAT (100 U/mL), and the products were immediately analyzed by HPLC, as described above.

HPLC analyses of the products formed from 2-BA-BP in the presence of superoxide radical anion/hydroperoxyl radical

2-BA-BP (100 μM) was incubated with 200 μM hypoxanthine, xanthine oxidase (generating of a flux of $O_2^{\bullet-}$ of 1 μM/min), and 100 U/mL catalase in a phosphate buffer (100 mM, pH 7.4) containing 10% MeCN at room temperature for 50 min, and the products were immediately analyzed by HPLC, as described above. The flux of $O_2^{\bullet-}$ was determined by monitoring the cytochrome c reduction following the increase in absorbance at 550 nm (using molar absorption coefficients difference between the reduced and oxidized forms of 2.1×10^4 M^{-1} s^{-1}).[6] Hydroperoxyl radical ($HO_2^{\bullet}$), a protonated form of $O_2^{\bullet-}$ is present in acid-base equilibrium (pK_a = 4.8).[7]

HPLC analyses of the products formed from 2-BA-BP in the presence of singlet oxygen

2-BA-BP (100 μM) was incubated with 1 mM H_2O_2 and 50 μM HOCl in a phosphate buffer (100 mM, pH 7.4) containing 10% MeCN at room temperature for 30 min, and the products were immediately analyzed by HPLC, as described above. Under the conditions used, HOCl reacts predominantly with H_2O_2 to form $^1O_2$ within <1 min after mixing.[8]

HPLC analyses of the products formed from 2-BA-BP in the presence of hydroxyl radical

2-BA-BP (35 μM) was incubated with 1 mM H_2O_2 and 50 μM $FeSO_4$ in perchloric acid (0.1 mM) containing 0.1% MeCN at room temperature for 10 min, and the products were immediately analyzed by HPLC, as described above. Under the conditions used, Fe^{2+} reacts with H_2O_2 to form $^{\bullet}OH$ within 2 min after mixing.[9] We estimated that around 40% of the generated $^{\bullet}OH$ pool reacts directly with 2-BA-

BP, while the remaining $^{\bullet}OH$ pool is scavenged mostly by MeCN (~50%). Direct interaction is confirmed by observed significant decomposition of 2-BP-BA after incubation.

Inhibition of ABTS$^{\bullet+}$ formation

To examine the effects of 2-BA-BP and 4-BA-BP on this process, 200 µM ABTS in 100 mM phosphate buffer (pH 7.4) was mixed with 100 µM the boronate compound at room temperature, and then 0–80 µM $ONOO^-$ was added. After rapid mixing, absorbance of samples at 735 nm or absorption spectra were immediately recorded using a Jasco V–670 UV-vis–near infrared spectrophotometer (Jasco, Japan).

EPR spin-trapping experiments

Incubation mixtures used in spin-trapping experiments consisted of 100 µM benzoylphenylboronic acid (2-BA-BP or 4-BA-BP), 20 mM MNP in a phosphate buffer (100 mM, pH 7.4) containing dtpa (100 µM) and were rapidly mixed with bolus 80 µM $ONOO^-$. Where indicated, 10% 2-PrOH was added before adding $ONOO^-$. The reaction mixtures were subsequently transferred to an EPR capillary, and the spectra were registered at room temperature in a Bruker Magnettech ESR 5000 spectrometer. Instrument parameters were as follows: magnetic field, from 331.9 mT to 341.9 mT; sweep time, 41 s; modulation amplitude, 0.1 mT; modulation frequency, 100 kHz; microwave power, 20 mW. The spectra shown were the average of 10 scans.

Solid phase extraction

For solvent exchange from aqueous to acetonitrile, solid phase extraction (SPE) cartridge (Thermo Fisher, SOLA HRP, 10 mg/mL) was firstly washed with 1 mL of MeCN, followed by 1 mL of water. The reaction mixture (0.8 mL) was applied to the SPE column, which was then washed with 1 mL of water. The columns were dried in vacuum for 5 min, followed by 30 min under air at room temperature, and again in vacuum for 15 min. Then, the oxidation products were eluted with 0.8 mL of dry MeCN, collected, and analyzed by HPLC and fluorimetry.

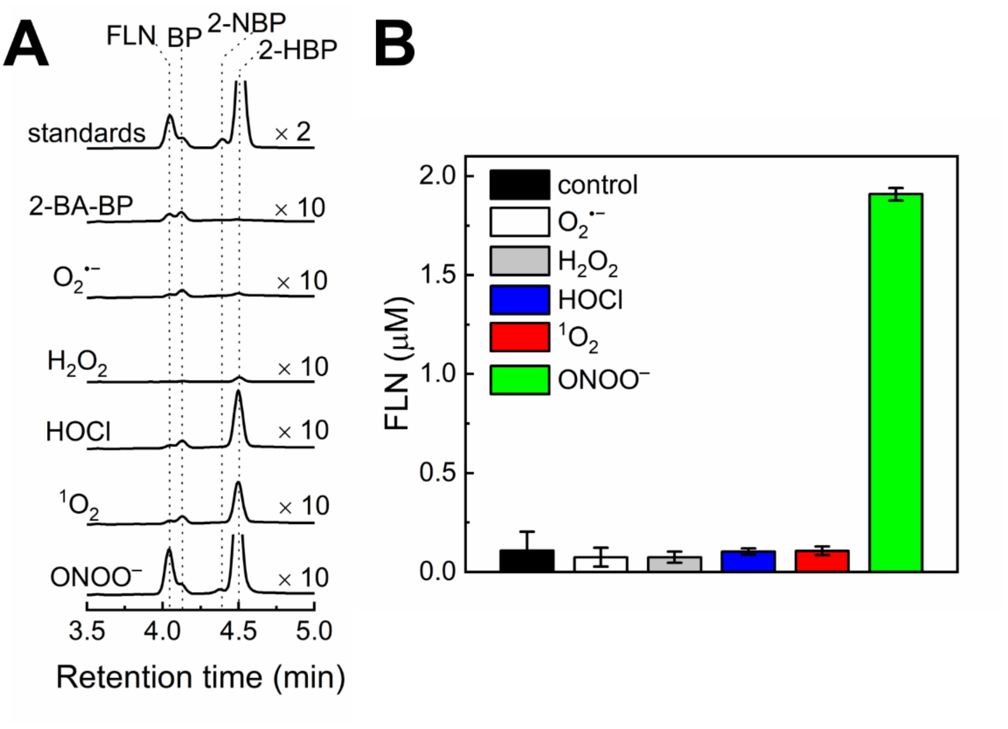

Figure S1. Analysis of FLN formation from 2-BA-BP in the presence of selected oxidants. (A) HPLC chromatograms obtained after the incubation of 2-BA-BP (100 µM) alone or in the presence of superoxide (1 µM $O_2^{\bullet-}$/min, 50 min), H_2O_2 (1 mM, 30 min), HOCl (50 µM, 30 min), H_2O_2 (1 mM) + HOCl (50 µM) to produce $^1O_2$ (30 min), or $ONOO^-$ (50 µM) in a phosphate buffer (100 mM, pH 7.4) containing 10% MeCN. The concentration of 2-BA-BP and 2-HBP standards was 100 µM, and the concentration of FLN, BP, and -NBP standards was 5 µM. (B) Quantitative analyses of the FLN formed in the systems shown in panel A.

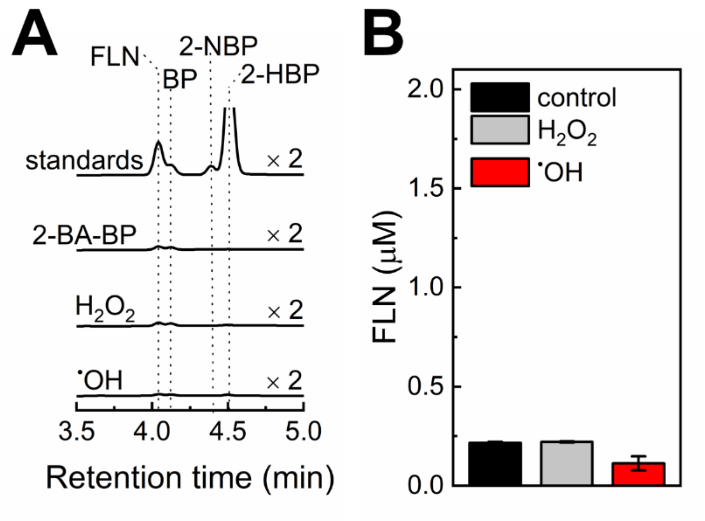

Figure S2. Analysis of FLN formation from 2-BA-BP in the presence of hydroxyl radical. (A) HPLC chromatograms obtained after the incubation of 2-BA-BP (35 µM) alone or in the presence of H_2O_2 (1 mM), or H_2O_2 (1 mM) and $FeSO_4$ (50 µM) in 0.1 mM perchloric acid (system producing $^{\bullet}OH$) containing 0.1% MeCN. HPLC traces were recorded after 10 min of incubation of 2-BA-BP with the oxidants. (B) Quantitative analyses of the FLN formed in the systems shown in panel A. The Y-scales in both panels are kept the same as in Figure S1, for a straightforward comparison.

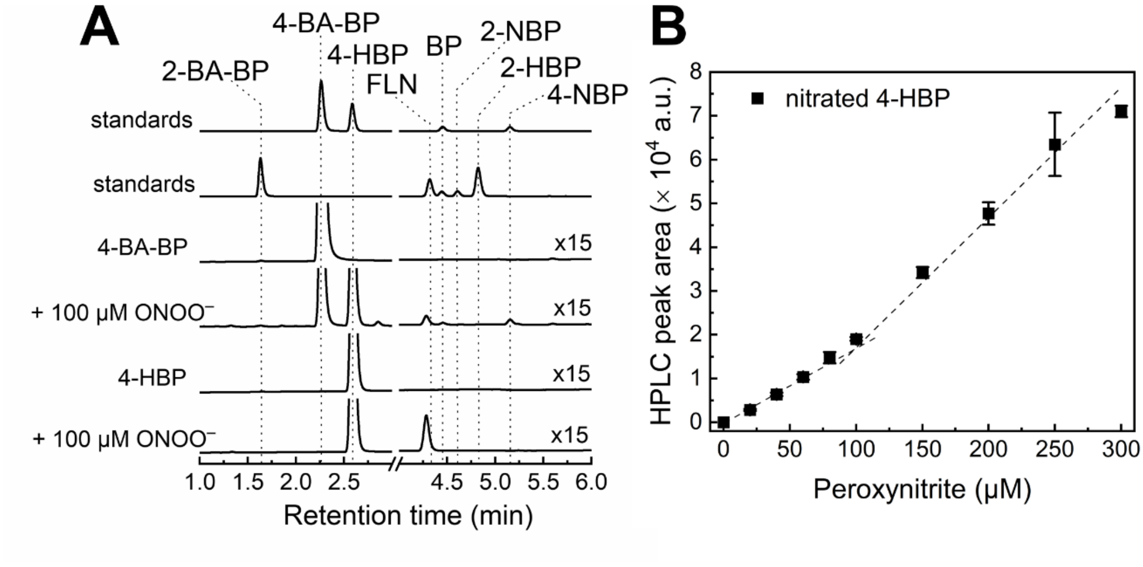

Figure S3. HPLC analyses of the products formed upon oxidation of the 4-BA-BP probe by ONOO⁻. (A) The HPLC chromatograms of the incubation mixtures containing 4-BA-BP (100 µM) or 4-HBP (100 µM) and ONOO⁻ (0, 100 µM) in a phosphate buffer (100 mM, pH 7.4 in the presence of 10 µM dtpa and 1% DMSO). The concentration of the 2-BA-BP, 2-HBP, 4-BA-BP, and 4-HBP standards was 100 µM, and the concentration of the FLN, BP, 2-NBP, and 4-NBP standards was 10 µM. (B) Nitrated 4-HBP formation during the ONOO⁻ reaction with 4-BA-BP in a phosphate buffer (100 mM, pH 7.4 in the presence of 10 µM dtpa and 1% DMSO). Data are means ± standard deviation of three independent experiments. The HPLC traces were collected after 5 min incubation of probe with oxidant using the absorption detector set at 254 nm.

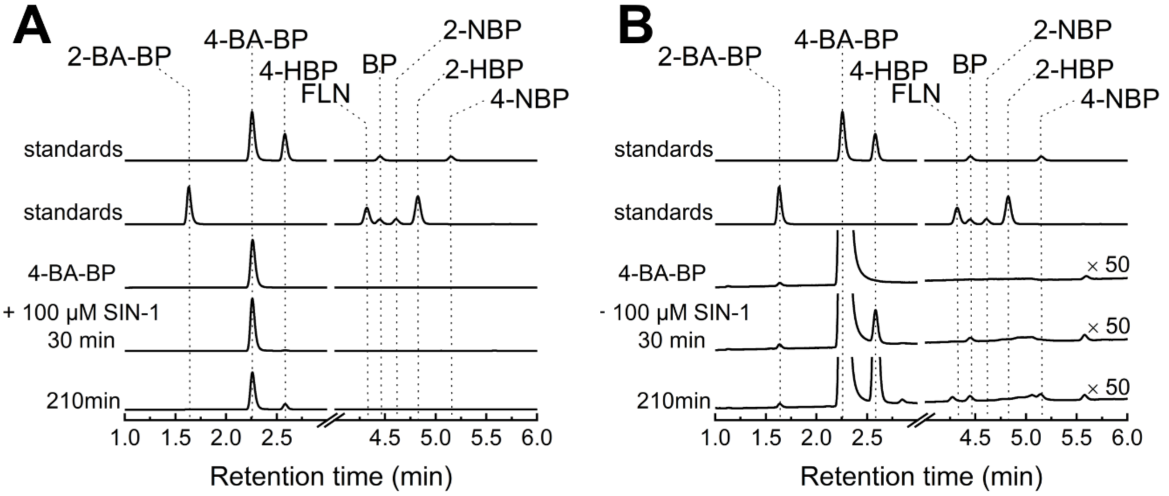

Figure S4. The chromatograms recorded upon analyses of products formed during oxidation of 4-BA-BP by *in situ*-generated ONOO⁻ from SIN-1. (A, B) Incubation mixtures consisted of 100 µM 4-BA-BP and 100 µM of SIN-1 in a phosphate buffer (100 mM, pH 7.4) containing dtpa (10 µM) and DMSO (1%). The HPLC traces were collected during 4-h incubation of 4-BA-BP with SIN-1 using the absorption detector set at 254 nm. The concentration of the 2-BA-BP, 2-HBP, 4-BA-BP, and 4-HBP standards was 100 µM, and the concentration of the FLN, BP, 2-NBP, and 4-NBP standards was 10 µM.

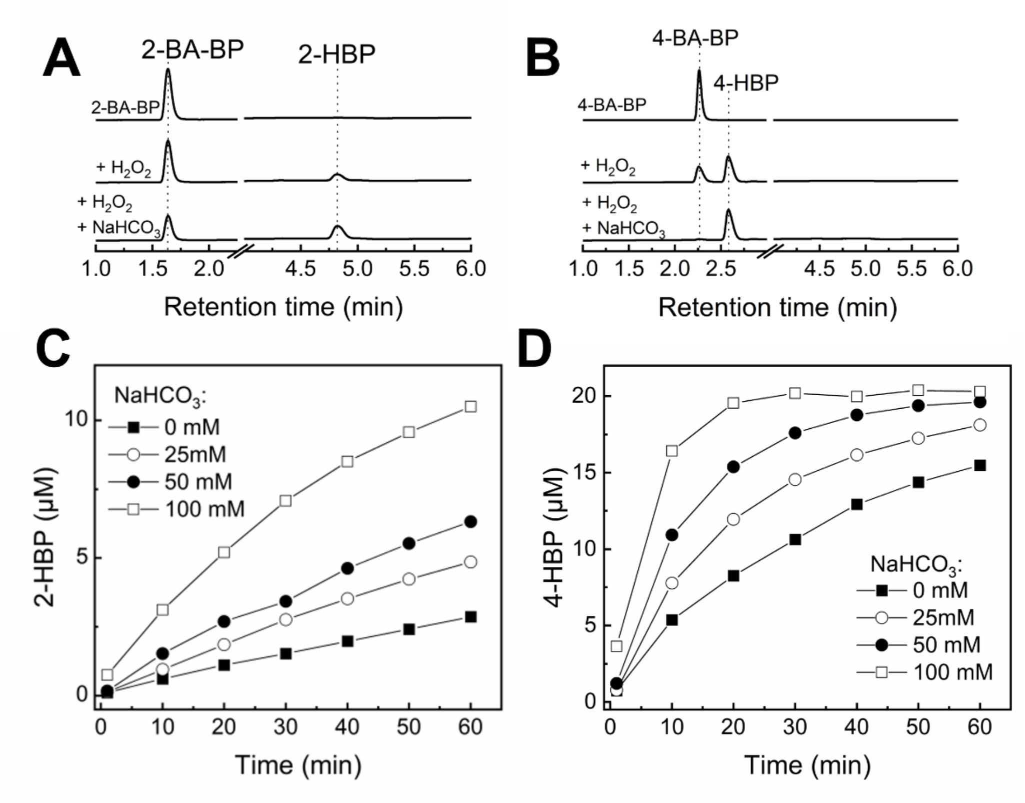

Figure S5. The effect of $NaHCO_3$ on 2-BA-BP and 4-BA-BP oxidation by H_2O_2. (A, B) HPLC traces of the products detected during the reaction between 2-BA-BP or 4-BA-BP (20 µM) and H_2O_2 (1 mM or 0.2 mM for 2-BA-BP and 4-BA-BP, respectively) in the absence and presence of $NaHCO_3$ (50 mM). (C, D) Dynamics of the formation of 2-HBP or 4-HBP in the presence of $NaHCO_3$ (0–100 mM). All solutions contained probe (20 µM), H_2O_2 (1 mM or 200 µM, for 2-BA-BP and 4-BA-BP, respectively), a phosphate buffer (100 mM, pH 7.4) with dtpa (10 µM), and DMSO (1%). The HPLC traces were collected using an absorption detector set at 254 nm.

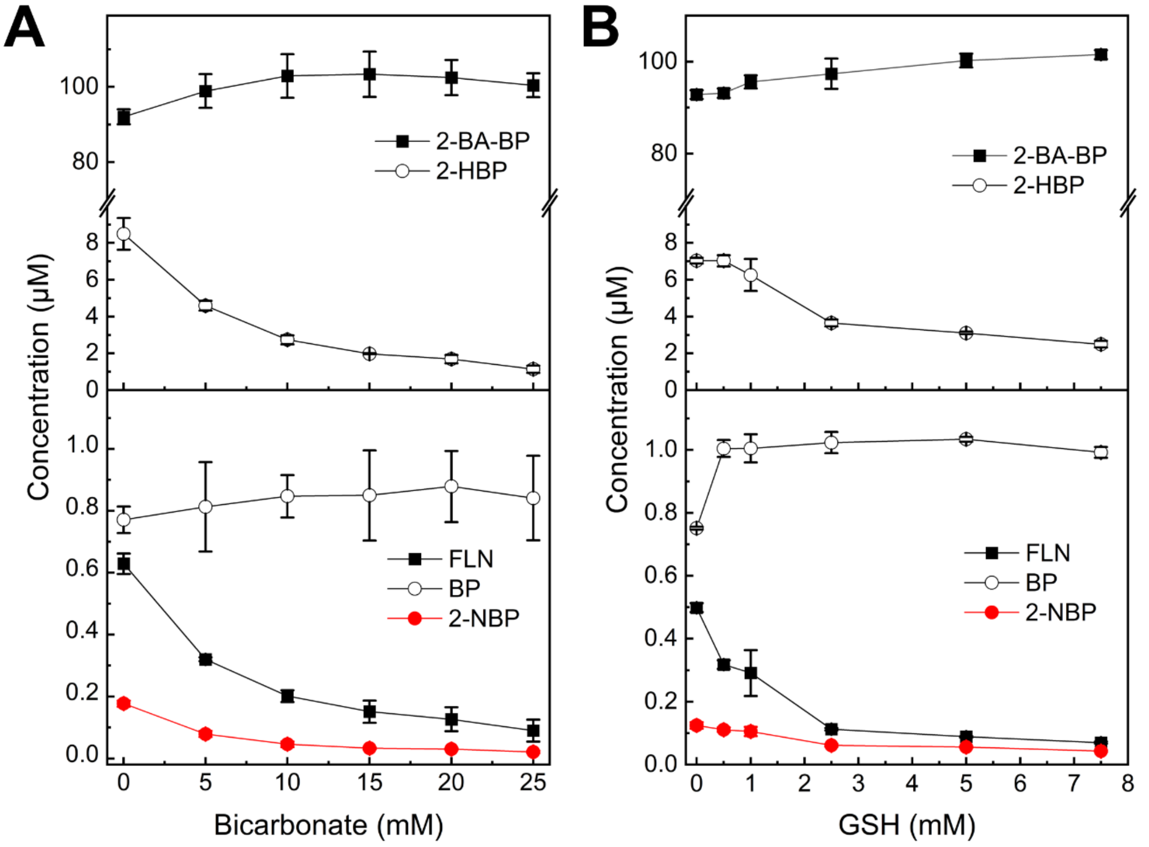

Figure S6. Product analyses for oxidation of 2-BA-BP in the presence of biologically relevant ONOO⁻ scavengers. Effects of (A) bicarbonate and (B) GSH on substrate depletion and major/minor product formation in the reaction between 2-BA-BP (100 μM) and ONOO⁻ (20 μM) in phosphate buffer (pH 7.4, 100 mM) containing dtpa (10 μM) and DMSO (1%). After a bolus addition of ONOO⁻, the reaction mixtures were analyzed by HPLC.

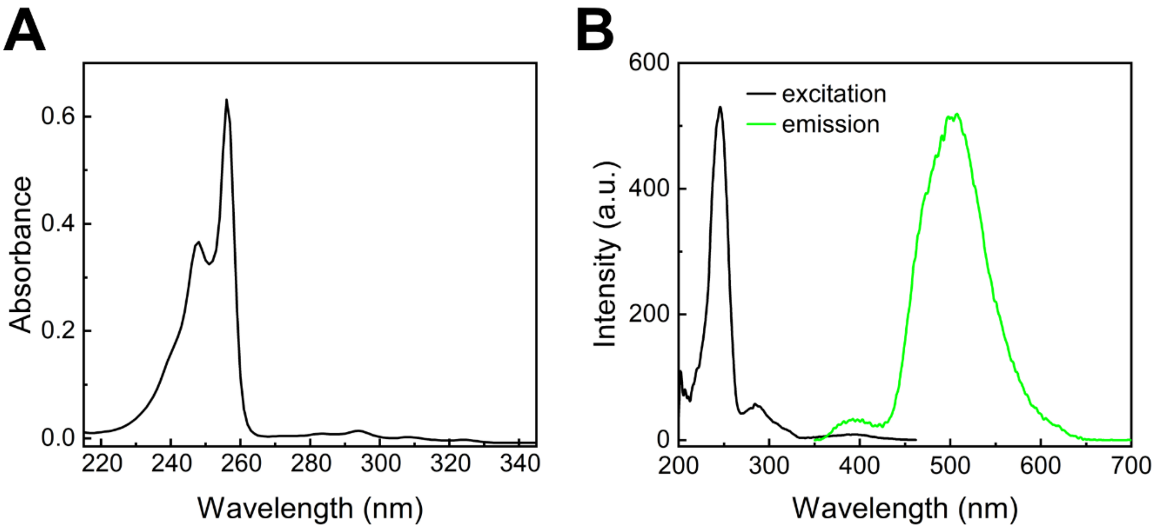

Figure S7. Spectroscopic characterization of FLN. (A) Electronic absorption, (B) excitation, and fluorescence spectra of FLN in MeCN. The concentration of compound was 5 μM in each case. λ_{ex}=248 nm, ex/em slits: 10/5 nm.

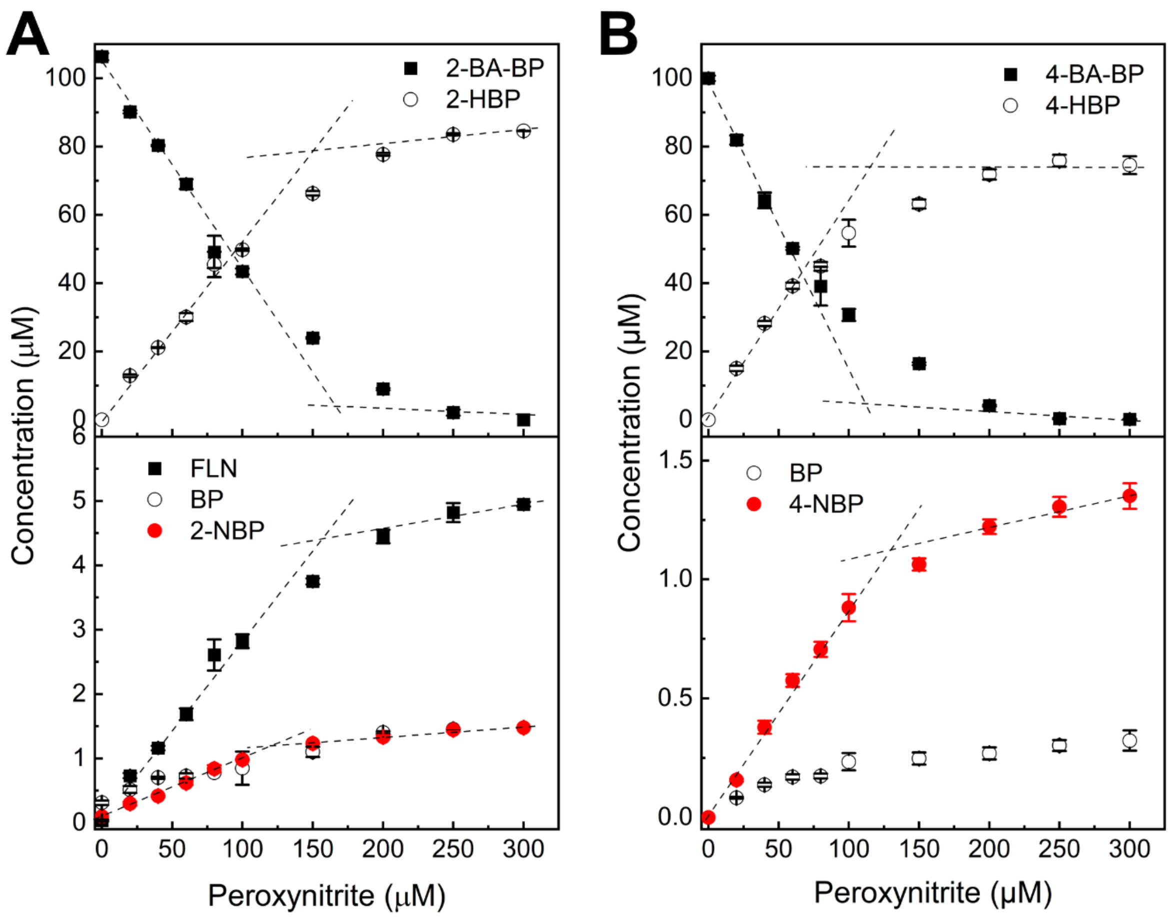

Figure S8. Reaction stoichiometry for oxidation of 2-BA-BP and 4-BA-BP by ONOO⁻. HPLC–based titration of (A) 2-BA-BP (100 μM) and (B) 4-BA-BP with ONOO⁻ (0–300 μM) in a phosphate buffer (100 mM, pH 7.4 in the presence of 100 μM dtpa, 10% MeCN and 0.1% DMSO in case of 2-BA-BP, and in the presence of 100 μM dtpa and 1% DMSO in case of 4-BA-BP). Data are means ± standard deviation of three independent experiments. ONOO⁻ was rapidly mixed with the probe using a vortex mixer and incubated for 5 min before HPLC analyses.

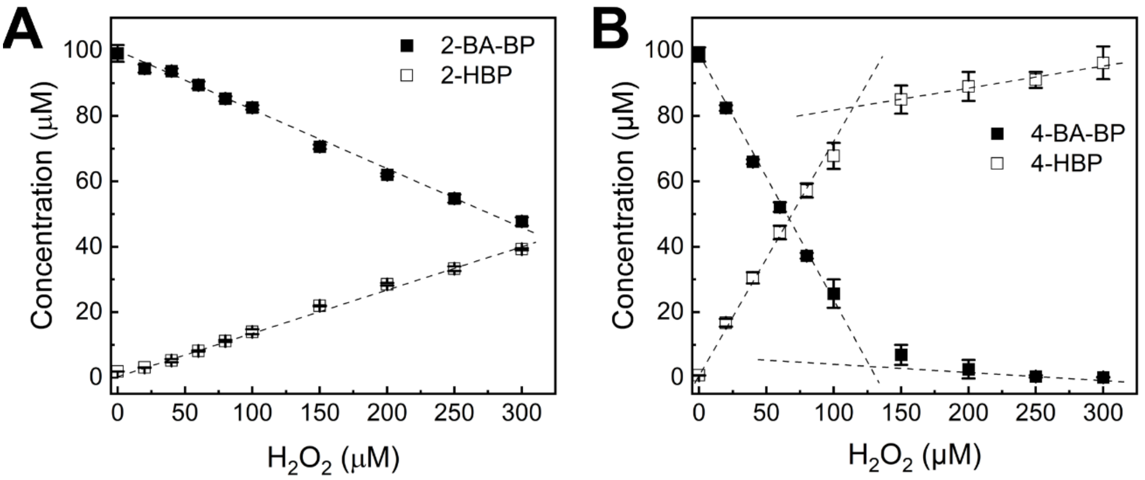

Figure S9. HPLC-based titration of 2-BA-BP and 4-BA-BP with H_2O_2. HPLC-based titration of (A) 2−BA-BP (100 μM) and (B) 4-BA-BP (100 μM) with H_2O_2 (0–300 μM) in a phosphate buffer (100 mM, pH 7.4) in the presence of 10 μM dtpa and 1% DMSO. Data are means ± standard deviation of three independent experiments. H_2O_2 was mixed with the probe using a vortex mixer and incubated for 24 h before HPLC analyses.

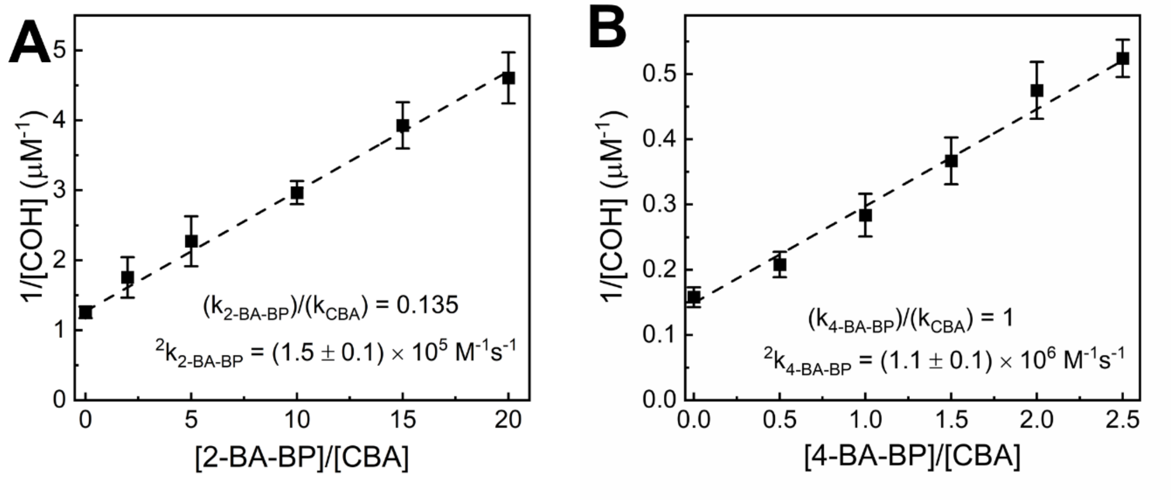

Figure S10. **Kinetics of the reaction of 2-BA-BP and 4-BA-BP with $ONOO^-$.** (A) The dependence of 1/[COH] on the [2-BA-BP]/[CBA] ratio after addition of 1 μM $ONOO^-$. $ONOO^-$ was rapidly mixed with a solution containing 5 μM CBA, 0–100 μM 2-BA-BP, and phosphate buffer (100 mM, pH 7.4 in the presence of 10 μM dtpa and 1% DMSO). (B) The dependence of 1/[COH] on the [4-BA-BP]/[CBA] ratio before addition of $ONOO^-$. $ONOO^-$ (10 μM) was rapidly mixed with a solution containing 20 μM CBA, 0–50 μM 4-BA-BP, and phosphate buffer (100 mM, pH 7.4 in the presence of 10 μM dtpa and 1% DMSO).The concentration of COH formed was determined by HPLC 5 min after addition of the oxidant.

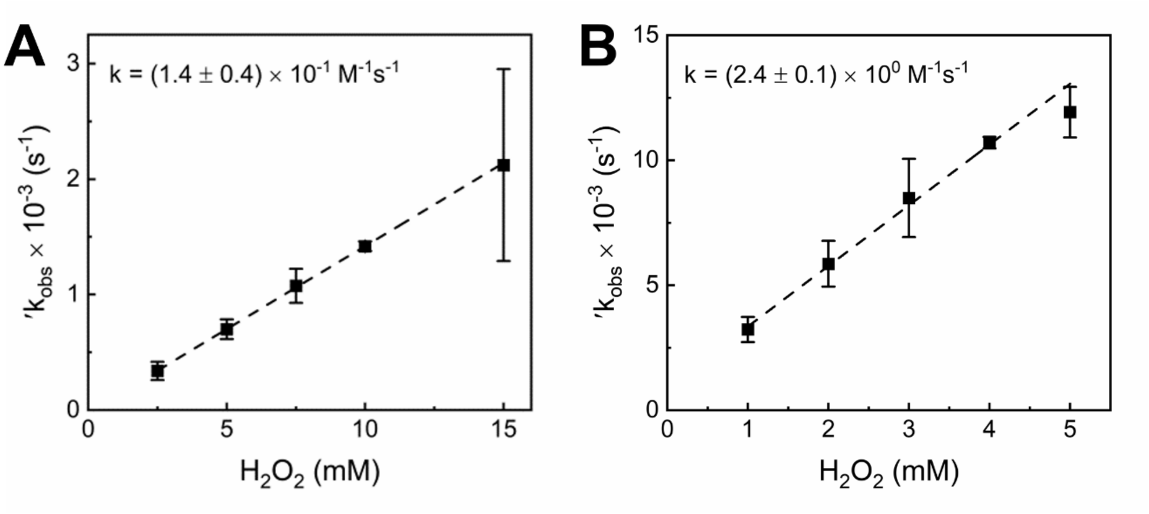

Figure S11. Kinetics of the reaction of 2-BA-BP and 4-BA-BP with H_2O_2. (A) Effect of H_2O_2 concentration on the pseudo-first-order rate constants ($'k_{obs}$) of the 2-BA-BP reaction with H_2O_2. Solutions consisted of the 2-BA-BP probe (100 μM), phosphate buffer (0.1 M, pH 7.4), dtpa (10 μM), DMSO (1%), and H_2O_2 (2.5–15 mM). (B) Effect of H_2O_2 concentration on the pseudo-first order rate constants ($'k_{obs}$) of the 4-BA-BP reaction with H_2O_2. Solutions consisted of the 4-BA-BP probe (100 μM), phosphate buffer (0.1 M, pH 7.4), dtpa (10 μM), DMSO (1%), and H_2O_2 (1–5 mM).

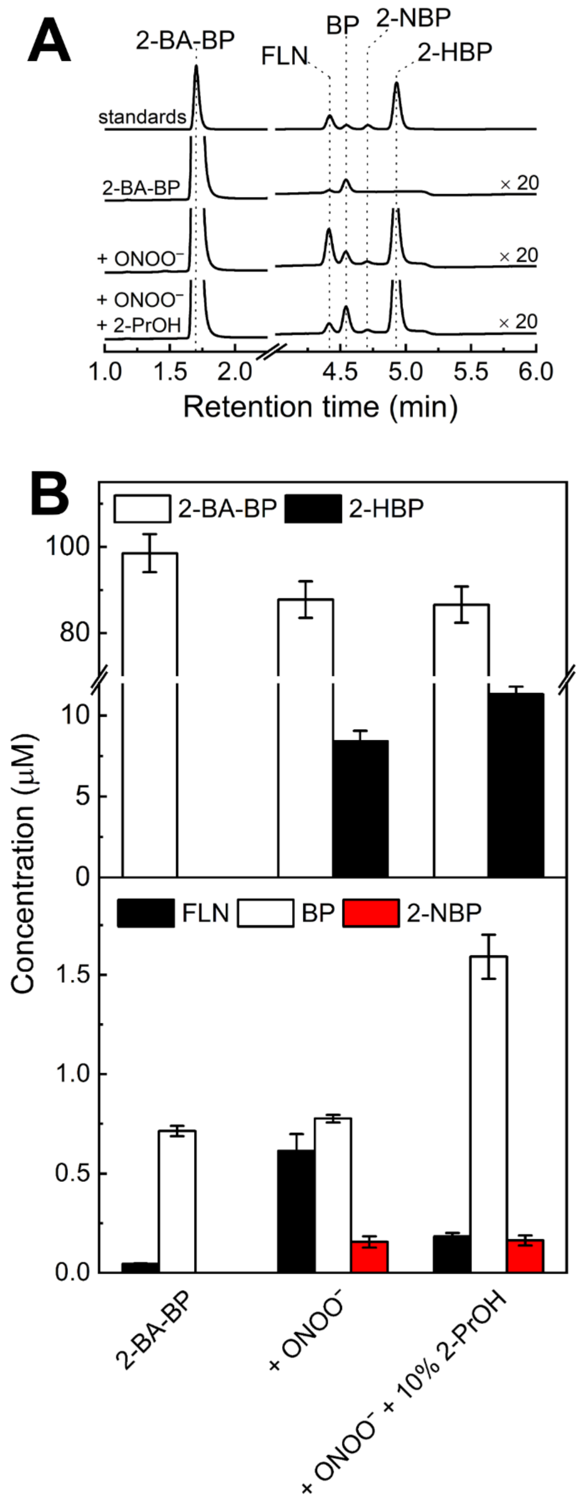

Figure S12. Effect of a phenyl radical scavenger, 2-PrOH, on products formed during the reaction between 2-BA-BP and ONOO⁻. (A) HPLC chromatograms of the products of reaction between 2-BA-BP and ONOO⁻ alone and in the presence of 10% 2-PrOH. (B) Quantitation of the products formed from the oxidation of 2-BA-BP by ONOO⁻ in the presence or absence of 10% 2-PrOH. Incubation mixtures consisted of 100 μM 2-BA-BP in phosphate buffer (pH 7.4, 100 mM) containing dtpa (10 μM), 1% DMSO, and 10% 2-PrOH (as indicated). After a bolus addition of ONOO⁻ (resulting in the 20 μM ONOO⁻ concentration in the sample), the reaction mixtures were analyzed by HPLC. The concentrations were determined based on the calibration curves obtained for authentic standards. Each bar represents the average value of three samples. The error bars represent standard deviations.

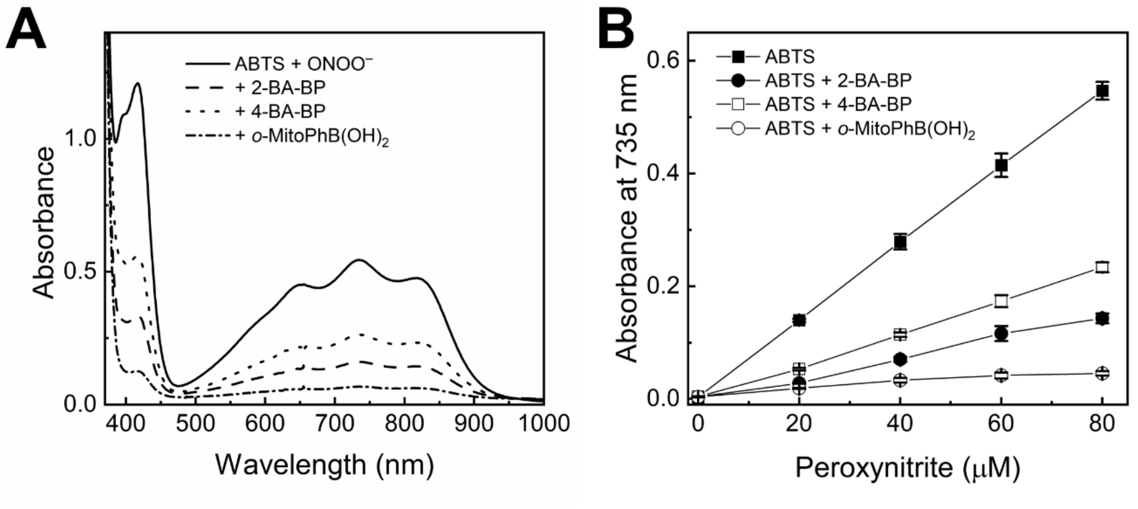

Figure S13. Inhibition of ONOO⁻–induced ABTS oxidation by 2-BA-BP, 4-BA-BP, and *o*-MitoPhB(OH)$_2$. (A) The absorption spectra of ABTS solutions after bolus addition of $ONOO^-$ (80 μM) in the absence and in the presence of 2-BA-BP, 4-BA-BP, or *ortho*-MitoPhB(OH)$_2$. Incubation mixtures consisted of 200 μM ABTS and 100 μM probe (as indicated) in phosphate buffer (pH 7.4, 100 mM) containing 1% DMSO. (B) The dependence of the absorbance at 735 nm on the $ONOO^-$ concentration in the presence of 2-BA-BP, 4-BA-BP, or *ortho*-MitoPhB(OH)$_2$ (100 μM). $ONOO^-$ (0−80 μM) was rapidly mixed with a solution containing 200 μM ABTS, 100 μM probe, and phosphate buffer (100 mM, pH 7.4, containing 1% DMSO). The absorption spectra were recorded immediately after a bolus addition of $ONOO^-$. Each point represents the average value of three samples. The error bars represent standard deviations.

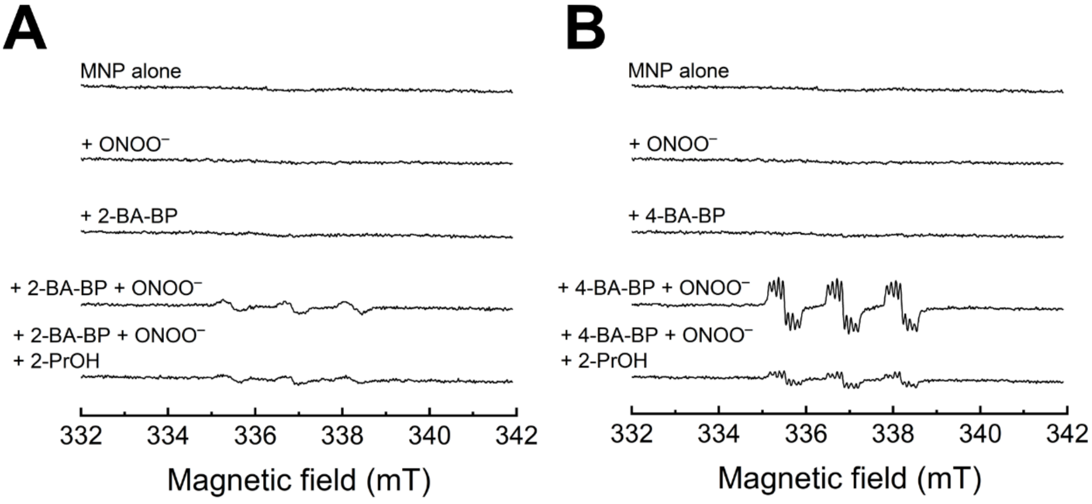

Figure S14. EPR spin trapping of the radical intermediates. MNP spin trap was used to trap the phenyl radical formed from the reaction between $ONOO^-$ and (A) 2-BA-BP or (B) 4-BA-BP. Incubation mixtures contained the following components: boronate probe (100 μM), $ONOO^-$ (80 μM), MNP (20 mM), 2.5% MeCN, and 1% DMSO in a phosphate buffer (100 mM, pH 7.4) containing dtpa (100 μM). Where indicated, 2-PrOH (10%) was added. The reaction mixture was transferred to an EPR cell immediately after adding bolus $ONOO^-$ to the reaction mixtures, and spectra were recorded at room temperature. The same signal intensity scale is used for panels (A) and (B).

^{1}H NMR and ESI MS spectra

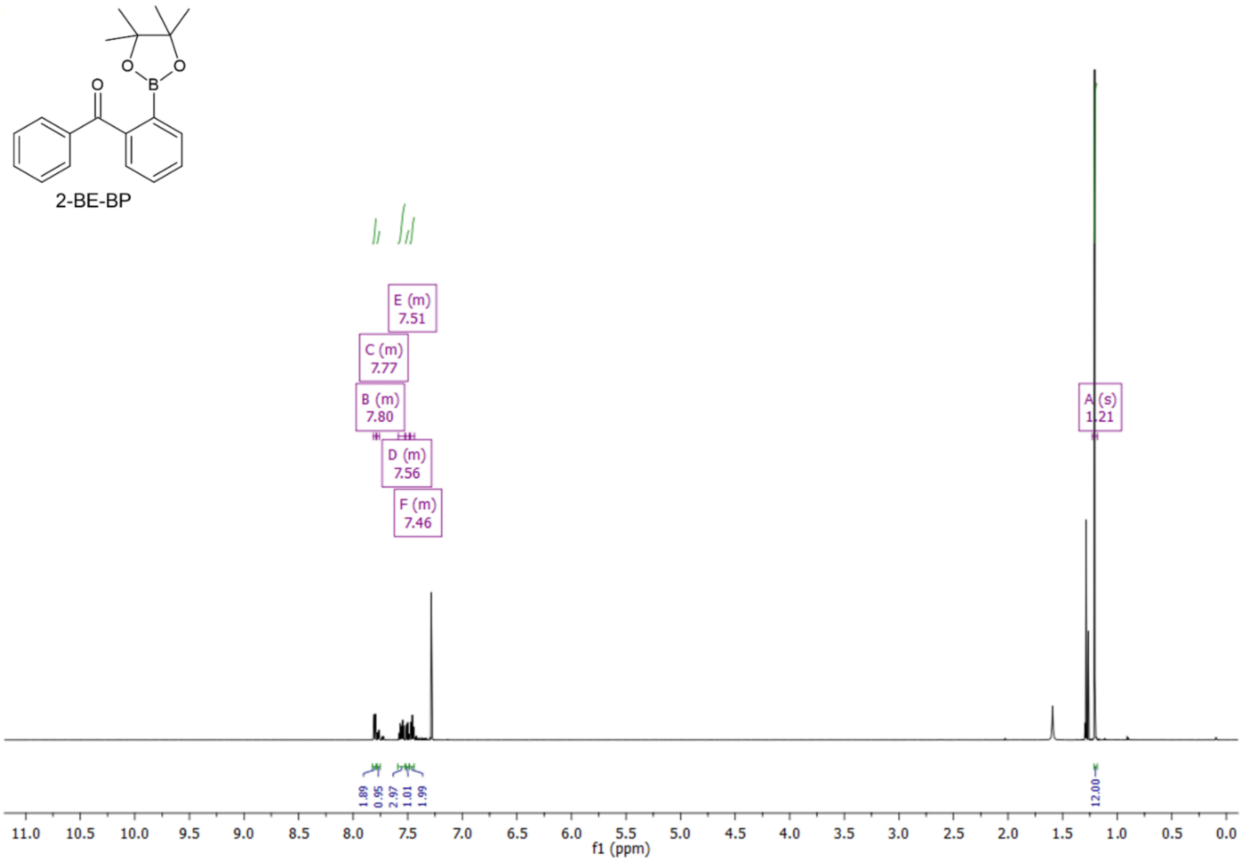

Figure S15. ^{1}H NMR spectrum of 2-BE-BP (700 MHz, $CDCl_3$). The signals attributable to impurities visible in the spectrum derive from BP and FLN products produced during microwave-assisted 2-BE-BP synthesis, as identified by the HPLC analyses.

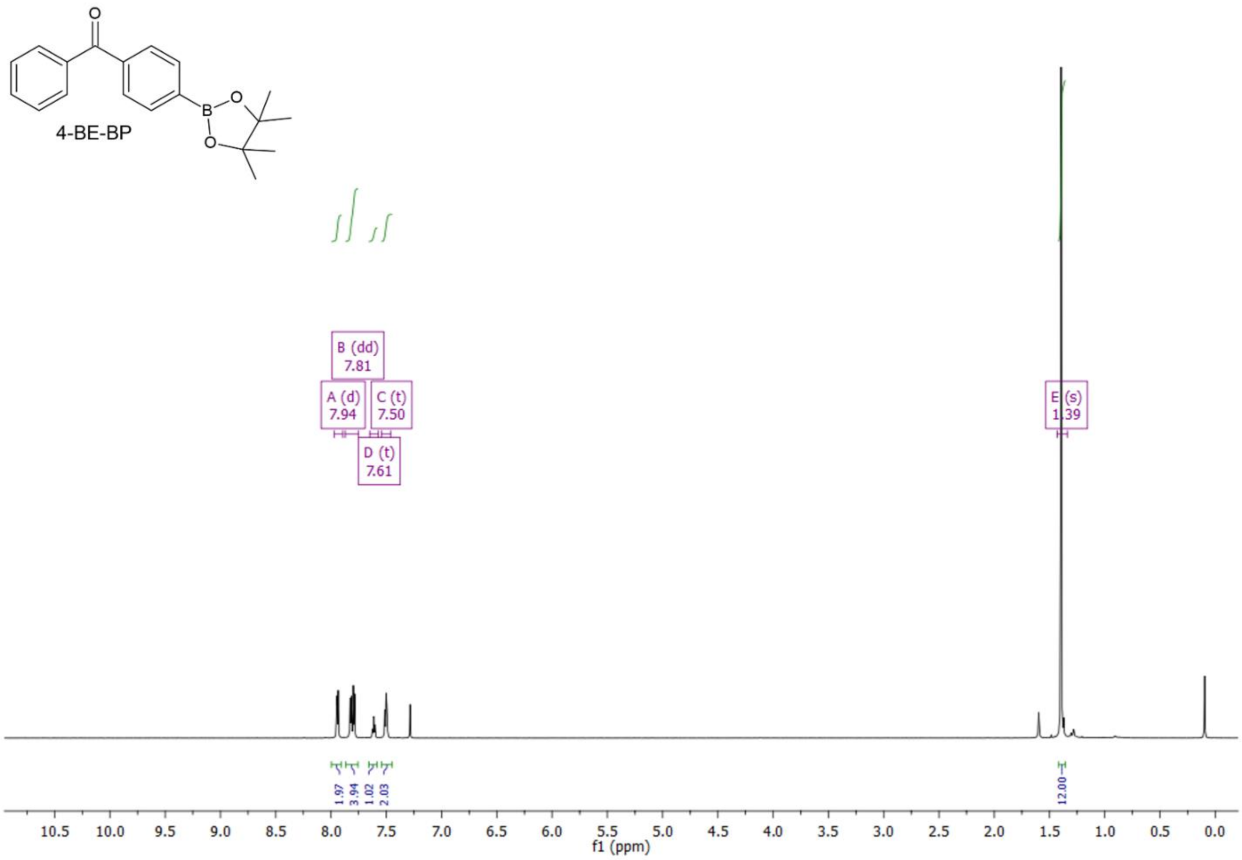

Figure S16. ^{1}**H NMR spectrum of 4-BE-BP (700 MHz,** $CDCl_3$**).**

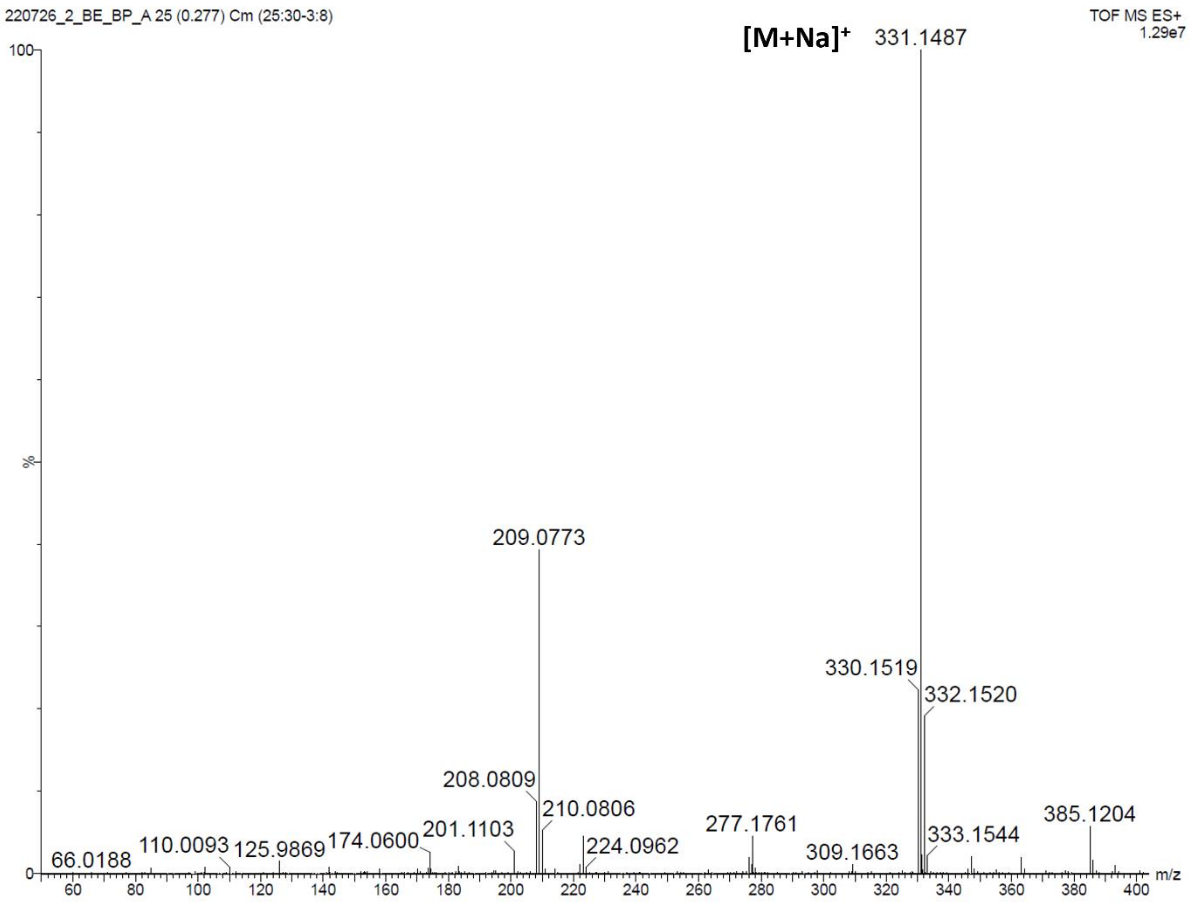

Figure S17. HRMS ESI spectrum of synthesized 2-BE-BP.

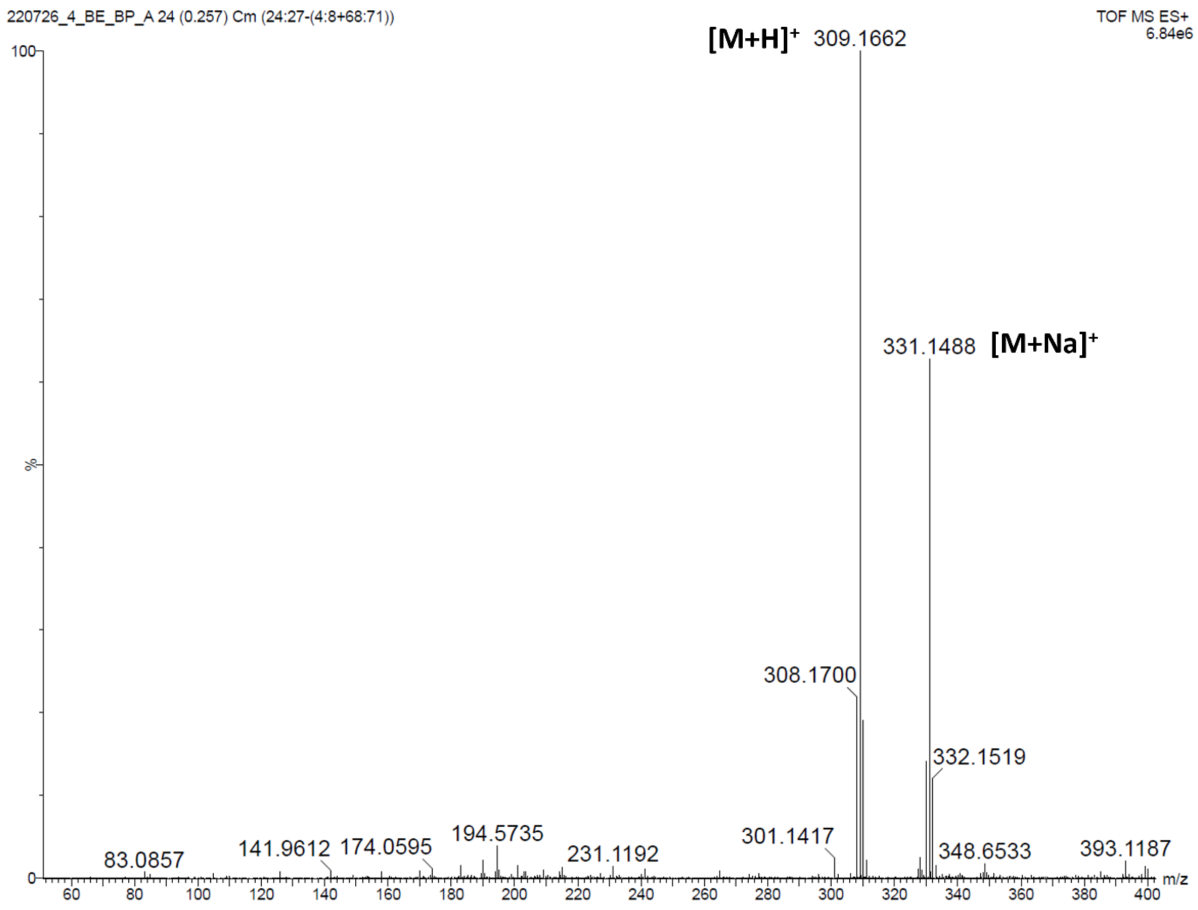

Figure S18. HRMS ESI spectrum of synthesized 4-BE-BP.

References

(1) Robinson, K. M.; Beckman, J. S. Synthesis of peroxynitrite from nitrite and hydrogen peroxide. *Methods Enzymol* **2005**, *396*, 207-214. DOI: 10.1016/S0076-6879(05)96019-9 From NLM Medline.

(2) Ma, J. M.; Ma, M. W.; Sun, L. H.; Zeng, Z.; Jiang, H. Synthesis, Herbicidal Evaluation, and Structure-Activity Relationship of Benzophenone Oxime Ether Derivatives. *J Chem-Ny* **2015**, *2015*. DOI: Artn 43521910.1155/2015/435219.

(3) Du, L.; Li, M.; Zheng, S.; Wang, B. Rational Design of a Fluorescent Hydrogen Peroxide Probe Based on the Umbelliferone Fluorophore. *Tetrahedron Lett* **2008**, *49* (19), 3045-3048. DOI: 10.1016/j.tetlet.2008.03.063 From NLM PubMed-not-MEDLINE.

(4) Zielonka, J.; Podsiadly, R.; Zielonka, M.; Hardy, M.; Kalyanaraman, B. On the use of peroxy-caged luciferin (PCL-1) probe for bioluminescent detection of inflammatory oxidants and - Identification of reaction intermediates and oxidant-specific minor products. *Free Radical Bio Med* **2016**, *99*, 32-42. DOI: 10.1016/j.freeradbiomed.2016.07.023.

(5) Rios, N.; Piacenza, L.; Trujillo, M.; Martinez, A.; Demicheli, V.; Prolo, C.; Alvarez, M. N.; Lopez, G. V.; Radi, R. Sensitive detection and estimation of cell-derived peroxynitrite fluxes using fluorescein-boronate. *Free Radic Biol Med* **2016**, *101*, 284-295. DOI: 10.1016/j.freeradbiomed.2016.08.033 From NLM Medline.

(6) Massey, V. The microestimation of succinate and the extinction coefficient of cytochrome c. *Biochim Biophys Acta* **1959**, *34*, 255-256. DOI: 10.1016/0006-3002(59)90259-8 From NLM Medline.

(7) Benon H., B. J., Cabelli D.E., Arudi R. L., Ross A.B. . Reactivity of HO2/O−2 Radicals in Aqueous Solution. *J. Phys. Chem. Ref. Data* **1985**, *14*, 1041–1100. DOI: 10.1063/1.555739.

(8) Held A.M., H. D. J., Hurst J.K. . Mechanisms of chlorine oxidation of hydrogen peroxide. *Journal of the American Chemical Society* **1978**, *100* (18), 5732–5740. DOI: 10.1021/ja00486a025.

(9) T.J., H. THE RATE CONSTANT OF THE REACTION BETWEEN FERROUS IONS AND HYDROGEN PEROXIDE IN ACID SOLUTION. *Canadian Journal of Chemistry* **1957**, *35* (5), 428-436. DOI: 10.1139/v57-062.
